# Reconfiguration of Multiphase Coacervate Droplets Into Self‐Regulated Nested Artificial Cells

**DOI:** 10.1002/anie.9334343

**Published:** 2026-04-10

**Authors:** Zhuping Yin, Rui Sun, Jingxin Shao, Jan C. M. van Hest, Stephen Mann

**Affiliations:** ^1^ Centre for Protolife Research and Centre for Organized Matter Chemistry, School of Chemistry University of Bristol Bristol UK; ^2^ Bio‐Organic Chemistry, Departments of Biomedical Engineering and Chemical Engineering & Chemistry Institute For Complex Molecular Systems Eindhoven University of Technology Eindhoven Netherlands; ^3^ Max Planck‐Bristol Centre for Minimal Biology, School of Chemistry University of Bristol Bristol UK

**Keywords:** artificial cell, coacervate, nested coacervate vesicle

## Abstract

Dynamic sub‐compartmentalization and internal organization are important assets of living cells to control functional complexity. Mimicking these features in artificial cells provides a platform to effectively respond to external cues by changing internal structure, thereby emulating life‐like behavior. Here, we present a strategy to construct sub‐compartmentalized artificial cells by converting multiphase coacervate droplets (MCDs) into nested coacervate vesicles (NCVs), in which the outer host domain is electrostatically reconfigured into a continuous semipermeable shell, while the internal guest droplets are preserved. The generated artificial cells exhibit spatial segregation of coacervate constituents and encapsulated fluorescent dyes, enzymes, and gold nanoparticles, and remain morphologically stable under different conditions. The membranized artificial cells display artificial metabolic features by means of poly(*N*‐isopropylacrylamide) (PNIPAAm) synthesis and subsequent temperature‐dependent aggregation, leading to emergent behavior including self‐regulated photothermal transitions, feedback‐mediated photocatalysis, and spatiotemporal organization of internal cargoes. Overall, our approach establishes a robust artificial cell platform that combines sub‐compartmentalization with self‐regulating properties, integrating functionality with structural complexity.

## Graphical abstract

Living cells advance functionality through spatiotemporal sub‐compartmentalization and internal organization. Herein, we develop a self‐regulated artificial cell by transforming a membrane‐less multiphase coacervate droplet (MCD) into a membranized, structurally reinforced nested coacervate vesicle (NCV). In situ poly(*N*‐isopropylacrylamide) PNIPAAm synthesis and LCST‐triggered aggregation enable self‐regulated photothermal effects and feedback‐mediated photocatalysis, advancing autonomous synthetic protobiology.

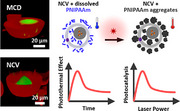

## 1 Introduction

Living cells orchestrate intricate processes such as gene expression, intra‐ and intercellular signalling, energy production, motility, and migration largely through the evolutionary development of distinct organelles (e.g., nuclei, mitochondria, cytoskeletons, biological condensates) that spatiotemporally organize cellular constituents and regulate metabolic pathways [1]. In the field of artificial cell research, introducing these organizational features by creating synthetic organelles within cell‐like compartments is therefore regarded as an essential approach to emulate life‐like behavior [2, 3]. To this end, artificial cells capable of executing spatially confined reactions, programmable signal release, self‐powered filament growth, and regulated gene expression have been realized through the formation of membranous subcompartments [3, 4, 5] or the self‐assembly of stimuli‐responsive, membrane‐less condensates [6, 7, 8, 9]. Furthermore, self‐assembled membranous artificial cells have been constructed and equipped with synthetic cytoskeletal structures using various building blocks (e.g., peptides, DNA, actin, and ParMRC system) [10, 11, 12, 13, 14, 15]. The cytoskeletons modulate interactions with synthetic membranes and internal components (e.g., plasmids, SUVs, and LUVs) [13, 14, 15], enabling responsive structural and functional adaptation (e.g., robustness, morphogenesis, spatiotemporally regulated localization, and transport of cargoes) [10, 11, 12]. Alternatively, complex coacervate droplets formed through associative liquid–liquid phase separation have emerged as promising templates for artificial cells [16]. These systems have attracted considerable interest due to their biologically relevant properties, such as spontaneous uptake and enrichment of biomolecular cargo from dilute environments, as well as their molecularly crowded and relatively hydrophobic interiors, which are conducive to enzyme‐mediated reactions [17, 18]. The functionality of coacervate‐based artificial cells has been also extended by creating synthetic organelles through in situ membranization [19, 20, 21] and incorporation of organelle‐like enclosed subcompartments [22, 23] or cytoskeleton‐like microstructures [24, 25], enabling enhanced structural robustness [20, 21], motility [21], morphogenesis [22, 24], and spatiotemporally regulated signalling processes [22, 23, 25].

Compared with the above‐described constructs, multiphasic coacervate droplets capable of intrinsic segregation of compositional and functional constituents [26, 27, 28, 29, 30, 31, 32, 33] offer a higher level of dynamic control over life‐like behavior. However, such membrane‐less liquid condensates suffer from issues such as structural instability and inadequate spatial segregation of bioactive components under out‐of‐equilibrium conditions, which hamper their applicability. Besides, coacervate systems capable of emergent behaviors (e.g., feedback loops) based on exploiting the dynamicity of their organelle‐like architectures to enable self‐induced compositional and functional adaptation remain scarce [22, 34].

**FIGURE 1:**
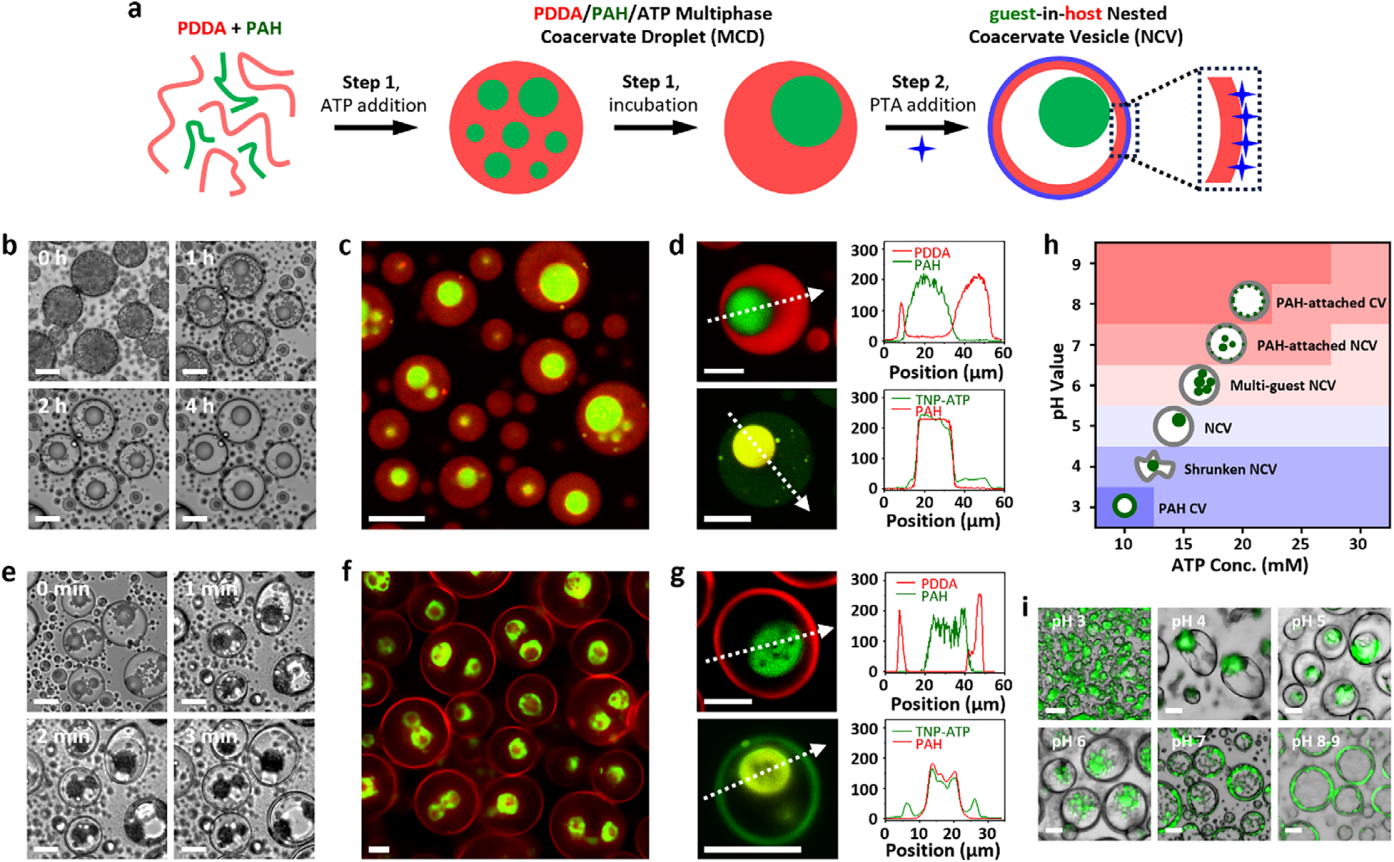
Formation of PDDA/PAH/ATP, MCDs, and NCVs. (a) Scheme showing a two‐step protocol to prepare MCDs and guest‐in‐host NCVs. Step 1: MCDs are generated by adding ATP to an aqueous mixture of PDDA (red line) and PAH (green line) to induce associative liquid–liquid phase separation. The MCDs contain dozens to hundreds of PAH/ATP‐rich guest droplets (green spheres) dispersed within a PDDA/ATP‐rich host phase (red sphere). After incubation under acidic conditions (pH < 5), the inner PAH/ATP droplets fuse to form 1–2 larger droplets. Step 2: PTA (blue star), a strongly anionic species that interacts with PDDA, is introduced to convert MCDs into guest‐in‐host NCVs. This transformation involves in situ membranization (blue) and electrostatically driven osmotic pressure changes at the droplet interface and interior, respectively, reconfiguring the PDDA/ATP host domain into a continuous shell of complexed polyelectrolytes while preserving the PAH/ATP guest droplets, owing to their higher matrix viscoelasticity. (b) Time‐dependent images of MCDs (PDDA/PAH/ATP, 20/10/12.5 mM) incubated at pH 4. (c, d) Fluorescence microscopy (c) and CLSM (d) images of MCDs after incubation, comprising RITC‐PDDA, FITC‐PAH, and nonfluorescent ATP (upper) or PDDA, RITC‐PAH and TNP‐ATP (bottom); line profiles indicate constituent distributions (d, right). (e) Time‐dependent images of MCDs undergoing morphological transition into NCVs in the presence of PTA (3 mM, pH 7). (f, g) Fluorescence microscopy (f) and CLSM (g) images of NCVs comprising RITC‐PDDA, FITC‐PAH, ATP, and PTA (upper) or PDDA, RITC‐PAH, TNP‐ATP, and PTA (bottom); line profiles indicate constituent distributions (g, right). (h) State diagram showing coacervate morphologies as a function of ATP concentration and pH, including PAH‐based coacervate vesicles (PAH CV), shrunken NCV, NCV, multi‐guest NCV, PAH‐attached NCV, and PAH‐attached CV. PTA, 3 mM. (i) A series of coacervate vesicles (PDDA/FITC‐PAH/ATP/PTA, 20/10/12.5/3 mM) prepared at different pH values (3–9). Scale bars, 20 µm.

To address these limitations, we have developed a strategy to convert a multiphasic coacervate system comprising poly(diallyl dimethylammonium chloride) (PDDA), poly(allylamine hydrochloride) (PAH), and adenosine triphosphate (ATP) into structurally reinforced nested artificial cells via the addition of a polyoxometalate agent, sodium phosphotungstate (PTA) [35, 36]. This process transforms the outer PDDA/ATP coacervate phase into a continuous shell of electrostatically complexed polyelectrolytes (membrane‐like host domain), while preserving the inner PAH/ATP coacervate droplets (organelle‐like guest domain) in an internal aqueous phase (Figure 1a). Although nested microstructures have previously been obtained through reconfiguration of multiphasic coacervates [37], these constructs lack a condensed outer membrane and exhibit limited sub‐compartmental segregation, making it challenging to construct artificial cells with organelle‐like features. Here, our nested artificial cells display well‐defined membrane‐ and organelle‐like domains capable of separately immobilizing distinct bioactive cargoes (e.g., glucose oxidase (GOx) and gold nanoparticles). The artificial cell is structurally reinforced during reconfiguration and exhibits enhanced stability under harsh conditions (salted or proteolytic), thereby supporting metabolism‐like processes under variable environments. By initiating a GOx/gold nanoparticle‐mediated host‐to‐guest signalling cascade initiated with glucose, the nested artificial cells induce polymerization of *N*‐isopropylacrylamide (NIPAAm). As a result, artificial cells display emergent behaviors, including self‐regulated photothermal transitions, feedback‐mediated photocatalysis, and spatiotemporal regulation of encapsulated cargoes. Taken together, this work introduces a facile strategy to generate robust sub‐compartmentalized artificial cells capable of self‐induced compositional development and functionality, offering a versatile platform for advancing synthetic protobiology.

## 2 Results and Discussion

Complex multiphase coacervate droplets (MCDs) derived from associative liquid‐liquid phase separation were prepared by mixing negatively charged adenosine 5′‐triphosphate disodium salt hydrate (ATP, Mw = 551) with positively charged PDDA (Mw = 100–200 kDa, 20 mM) and PAH (Mw = ca. 50 kDa, 10 mM) under acidic conditions (pH value ca. 4.5) while vortexing (1000 rpm, 30 s) (Figure 1a). Optical microscopy images indicated that the generated complex coacervate droplets were spherical in shape with a diameter of 2–35 µm (mean = 10.9 ± 8.0 µm) and initially contained dozens to hundreds of PAH/ATP guest droplets (Figures 1b and S1). The guest droplets gradually fused to form larger PAH/ATP coacervate droplets (mean diameter = 8.3 ± 5.3 µm) within several hours, while the host PDDA/ATP coacervate droplets retained their size and shape (Figures 1a,b and S1). This resulted in the formation of MCDs with a well‐defined spatial segregation of components, in which PDDA (RITC‐labelled) and PAH (FITC‐labeled) polymers mostly self‐organized within the outer (host) and inner (guest) phases, respectively (Figures 1c,d and S2). CLSM images and SEM‐EDS mapping demonstrated that the negatively charged ATP molecules were more concentrated within the PAH‐rich guest domains of the MCD constructs (Figures S3 and S4). Because of the pH‐dependent protonation of ATP and PAH but not PDDA and the difference in PDDA/ATP and PAH/ATP matrix viscoelasticity (Figure S5), a continuum of coacervate droplets with different morphologies and numbers of guest domains was produced by altering composition and pH values (Figure S6). Single phasic PAH/ATP‐based coacervate droplets (*ζ* = + 20.1 mV, diameter = 2–10 µm) were generated at pH 3.0 and low ATP concentration (< = 10 mM). By increasing pH (> = 4) or ATP concentration (> = 12.5 mM), PDDA/PAH/ATP‐based multiphasic coacervate droplets with increasing numbers of guest domains were generated. The structures varied from single or double guest coacervates within the host (pH = 4–5, ATP = 10–30 mM) to multiple smaller guests (pH = 8–9, ATP = 10–30 mM).

In line with previous reports [27, 29], the PAH/ATP guest domains of the MCDs exhibited stronger compositional association than the PDDA/ATP host domains, corresponding to higher salt tolerance and matrix viscoelasticity (Figures S7 and S8). Upon addition of PTA (3 mM, pH 7)—a polyanion with strong affinity for cationic polyelectrolytes [35, 36] —the host domains of the MCD constructs were reconfigured within minutes into a continuous, membrane‐bound and PDDA‐rich shell of electrostatically complexed polyelectrolytes (coacervate vesicle), while the PAH‐rich organelle‐like guest domains were preserved, regardless of the formation of small water‐filled pockets during the out‐of‐equilibrium process [38]. During this PTA‐induced reaction–diffusion process [39], transient nonspherical intermediate morphologies were occasionally observed (1–2 min), which gradually swelled to spherical structures upon completion of reconfiguration (Figure 1e,f). The guest‐in‐host NCVs swelled (4–42 µm, mean = 19.2 ± 9.8 µm) by ingress of water (Figures 1a,e and S1). Fluorescence microscopy and CLSM indicated that the spatial distribution of RITC‐labeled PDDA, FITC‐labeled PAH, and ATP was sustained (Figures 1f,g, S2, and S4). SEM‐EDS mapping confirmed that PTA predominantly interacted with the host domain during reconfiguration, as indicated by enrichment of tungsten signals on the NCV shell (Figure S3). This was consistent with the shift in zeta potential (*ζ*) from + 5.5 mV for MCDs to –34.3 mV for NCVs at pH ca. 6.5 (Figure S9). By changing pH and ATP concentration, a variety of membrane‐bounded nested microstructures were generated through PTA‐induced reconfiguration of the MCDs (Figure 1h,i), including osmotically shrunken or swollen nested and non‐nested structures formed under different conditions. Based on these observations, we established a reproducible protocol for producing NCVs spherical in shape and incarcerating 1–5 guest domains; this involved the initial generation of MCDs composed of 20/10/12.5 mM PDDA/PAH/ATP, followed by incubation at pH 4.0 for >12 h, subsequent adjustment of the pH to 6.5, and addition of PTA (3 mM, pH 6.5).

**FIGURE 2:**
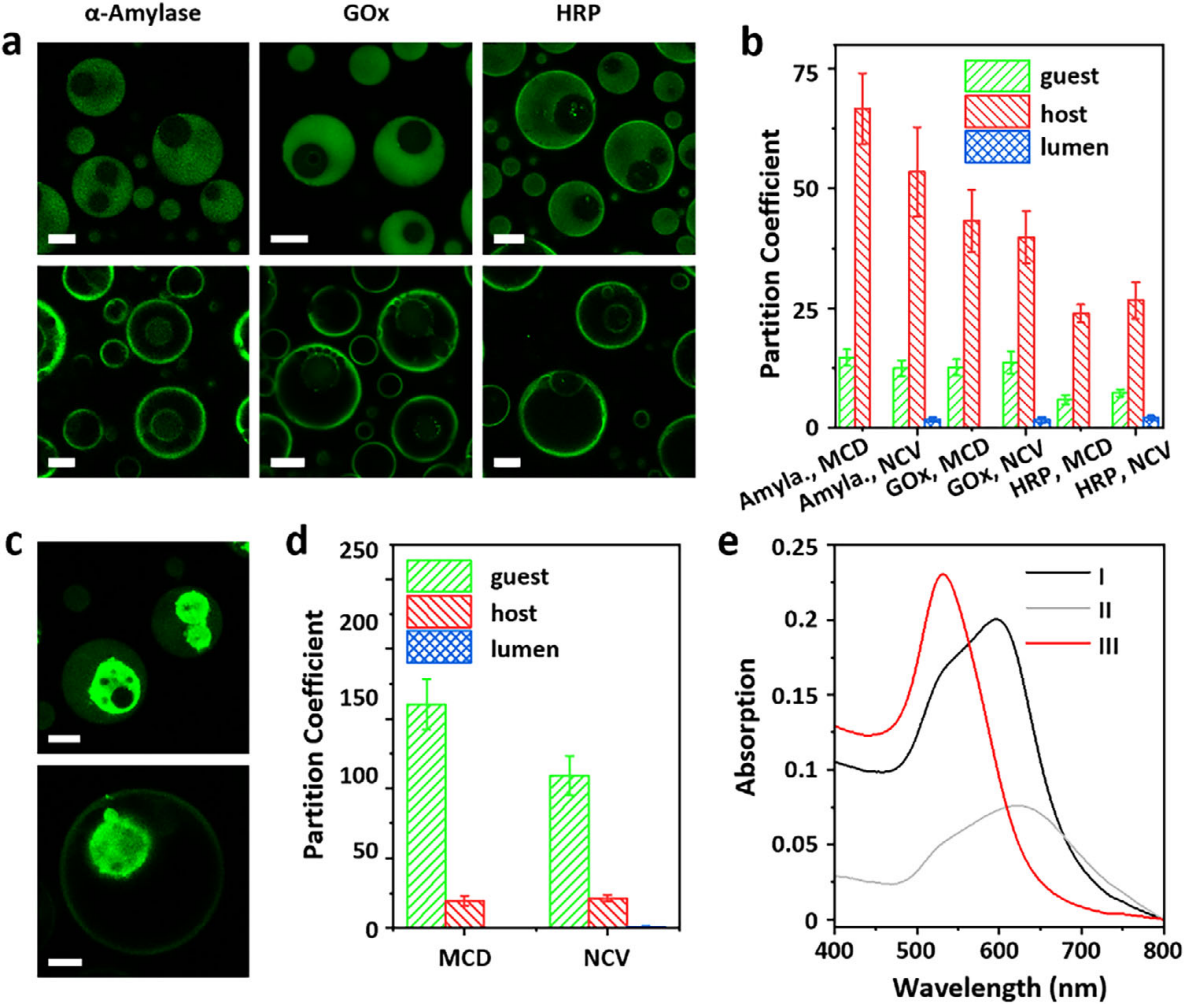
Spatial confinement of catalytic elements within the MCD and NCV constructs. (a) CLSM images of MCDs (top) and NCVs (bottom) loaded with FITC‐α‐amylase, FITC ‐glucose oxidase (FITC‐GOx), and FITC‐horse radish peroxidase (FITC‐HRP). FITC‐enzymes, ca. 0.05 mg/mL. (b) Partition coefficients of FITC‐enzymes in (a). (c) CLSM images of MCDs (top) and NCVs (bottom) loaded with FITC‐gold nanoparticles (20 nm). (d) Partition coefficients of FITC‐gold nanoparticles in (c). Partitioning was quantified as ratios of green fluorescence intensity (gray values) between the host or guest condensate, or lumen of NCV, and exterior diluted phase, and measured using Image J software. FITC‐gold nanoparticles, OD_520_ = ca. 0.05. Error bars: Data were obtained from > 30 MCDs or NCVs in 3 CLSM images (mean ± SD). (e) Absorption spectra of gold nanoparticles released from MCDs or NCVs upon dissociation with NaCl (1 M) and HCl (0.5 M). Samples were incubated at R.T. and pH 6.5 for 1 or 7 days. Gold nanoparticles, OD_520_ = ca. 0.25. Group I: MCD, 1 day; Group II: MCD, 7 days; Group III: NCV, 7 days. Condensate composition (a–e), PDDA/PAH/ATP, 20/10/12.5 mM; PTA, 0 or 3 mM. Scale bars, 20 µm.

The PTA/PDDA membrane of the NCV construct was morphologically continuous and semipermeable. No ingress of FITC‐labeled polysaccharides of different sizes (Mw = 4–250 kDa) was observed (Figure S10), but small‐molecule fluorescent dyes (Mw: calcein, 622.55 g/mol; resorufin, 213.19 g/mol) were membrane‐permeable (Figure S11). PTA‐induced electrostatic complexation was accompanied by increased membrane rigidity, enhanced viscoelasticity, and salt tolerance (Figures S7 and S8). Despite a reversal in surface charge (Figure S9), NCVs preserved the spatial organization of cargoes attained with the original MCDs, likely due to impeded electrostatic translocation of molecules within the reinforced matrix. This was demonstrated by the retained distributions of charged and neutral dyes (rhodamine 123, sulforhodamine B, Nile red) and polysaccharides (DEAE‐dextran, CM‐dextran) formed under electrostatic interactions (Figures S12 and S13). Similarly, FITC‐labeled enzymes such as amylase (*ζ* = –23.3 mV), GOx (*ζ* = −28.0 mV), and HRP (*ζ* = −8.1 mV) were efficiently sequestered in the MCDs (77.8%, 78.2%, and 68.1%, respectively) and stably retained in the NCVs (71.6%, 79.6%, and 65.6%, respectively) and localized primarily within the membrane‐like host domains, as verified by CLSM imaging and partition coefficients (Figure 2a,b). Similarly, the preferential localization of negatively charged gold nanoparticles (diameter = 20 nm, *ζ* = −18.9 mV) within the PAH/ATP‐enriched guest domains of the MCDs (guest: 89.5%, host: 7.3%) was preserved in the NCVs (organelle: 89.8%, membrane: 7.1%) after reconfiguration (Figure 2c,d). Encapsulated enzymes localized within the NCV membrane domain resisted salt‐induced release (200 mM) (Figure S14) and proteolytic degradation (Figure S15). UV–vis absorption spectra indicated that unaggregated nonfluorescent gold nanoparticles were released from the NCV organelle domain, while similar experiments with MCDs indicated that the 520 nm peak was redshifted, consistent with nanoparticle aggregation over a period of 7 days (Figure 2e). Collectively, these results demonstrate that NCVs inherit the organizational structure of the MCDs while providing structural reinforcement and cargo stabilization.

**FIGURE 3:**
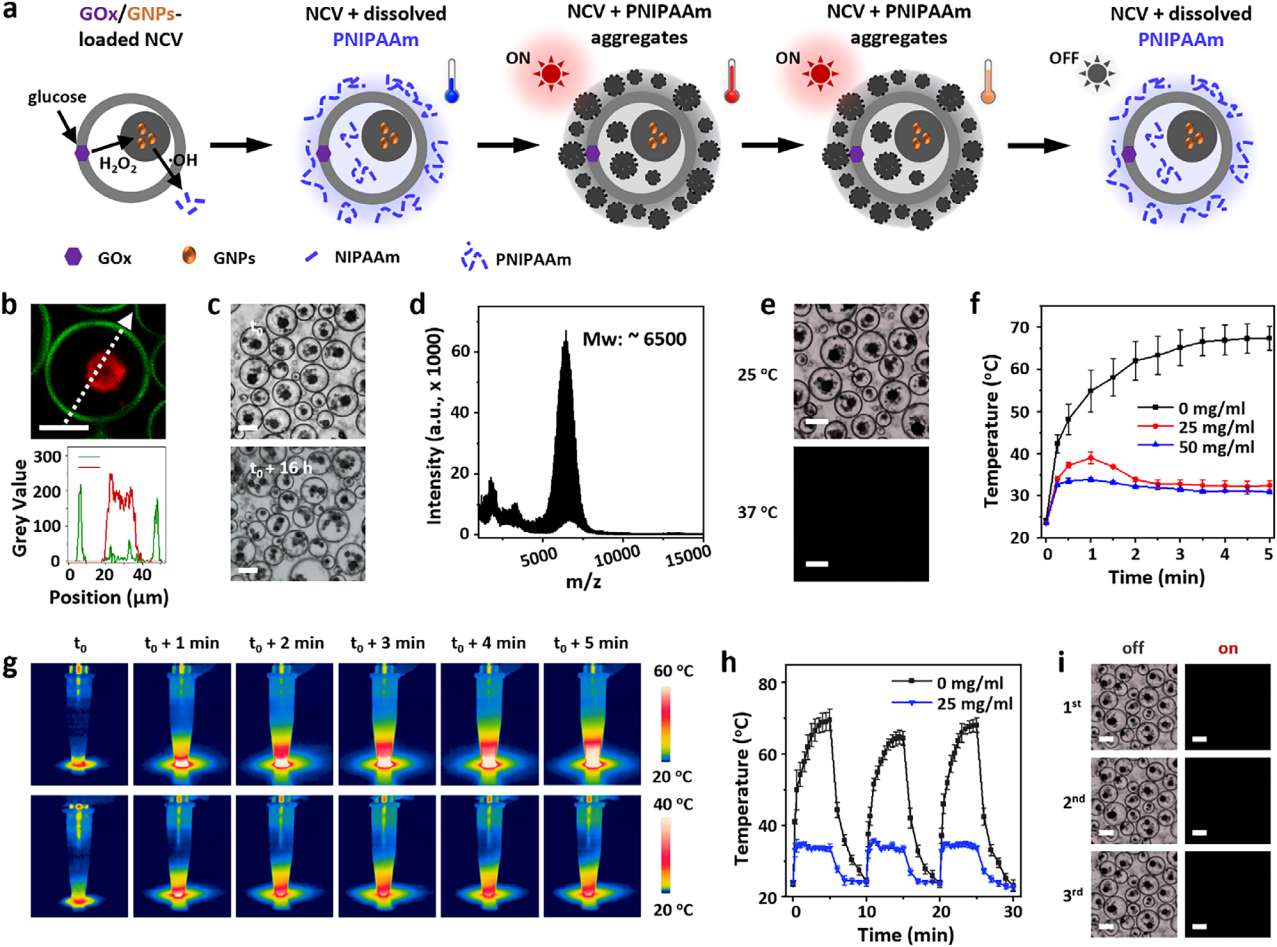
Dynamically regulated photothermal transition within NCV constructs. (a) Schematic illustration of a catalytic cascade process inducing polymerization in NCV constructs. Glucose activates GOx, spatially confined in the membrane of NCVs to produce H_2_O_2_, which diffuses to organelle‐localized gold nanoparticles, generating free radicals that trigger NIPAAm polymerization inside/outside NCVs to yield temperature‐responsive PNIPAAm. PNIPAAm aggregates block NIR light (808 nm) due to photothermal heating (LCST, ca. 32°C), leading to a self‐induced partially quenched photothermal transition under consistent NIR light irradiation. (b) CLSM images and line profiles of an NCV construct (bottom) loaded with FITC‐GOx (green) and RITC‐gold nanoparticles (red, 20 nm). (c) Bright field images of NCVs before (upper) and after (bottom) the glucose‐initiated PNIPAAm synthesis; [NIPAAm] ca 25 mg/mL, [glucose], ca. 10 mM. (d) MALDI‐ToF mass spectrum of PNIPAAm extracted from the NCV dispersion in (c), with a characteristic peak at ca. 6500 g/mol. (e) Bright field microscopy images of a colony of NCVs incubated at 25 and 37°C. At 37°C, PNIPAAm aggregates above the LCST, leading to scattering and therefore a dark image. (f) Photothermal transition plots of the NCV dispersions with different levels of NIPAAm polymerization (0, 25, and 50 mg/mL) and under NIR irradiation (808 nm, 6 W/cm^2^). (g) Corresponding thermal images of NCVs inducing NIPAAm polymerization at 0 (top) or 25 mg/mL (bottom). (h) Dynamic photothermal regulation of NCVs under cyclic ON–OFF NIR irradiation (ON at 0, 10, and 20 min; OFF at 5, 15, and 25 min). (i) Microscopy images of NCVs under repeated light cycling. From (**b**–**i**): PDDA/PAH/ATP, 10/10/12.5 mM; PTA, 3 mM. Error bars: *n* = 3 (repeated group of samples), mean value ± SD in (f,h). Scale bars, 20 µm.

Having established NCVs as membrane‐bound, cargo‐loaded and robust constructs, we next introduced a biomimetic function by integrating a GOx/gold nanoparticle‐mediated signalling pathway to induce compositional complexity (Figure 3a). In bulk, the cascade converted glucose into H_2_O_2_, which activated gold‐mediated radical formation and triggered in situ polymerization of *N*‐isopropylacrylamide (NIPAAm), yielding temperature‐responsive poly(*N*‐isopropylacrylamide) (PNIPAAm) within 24 h (Figure S16). This pathway was then compartmentalized in NCVs by localizing GOx in the PTA/PDDA membrane and the gold nanoparticles in the PAH/ATP organelle domains within the aqueous interior (Figure 3b). Upon glucose addition (ca. 10 mM), H_2_O_2_ production led to the generation of free radicals, thereby triggering NIPAAm polymerization (ca. 25 mg/mL monomer concentration) throughout the NCVs. The architecture of the compartments was preserved throughout the polymerization process (Figure 3c), underscoring the structural robustness of the nested artificial cells. In contrast, the MCD constructs were gradually deformed into vacuolated vesicles and ultimately became disrupted within 16 h, expelling the guest droplets due to the generated osmotic stress during in situ NIPAAm polymerization [40] (Figure S17). Disrupting this spatial signalling pathway by removing the organelle‐localized gold nanoparticles significantly impeded the polymerization process, resulting in only minimal polymer formation within the NCV microstructures (Figure S18).

Although the immobilized catalysts in the NCVs initially promoted spatially confined polymerization, efflux of the free radicals and the radicals generated by diffusive catalysts also induced PNIPAAm formation in the external medium over time (Figure 3d). Above the LCST (∼ 32°C), PNIPAAm therefore aggregated both within the NCVs and in the medium, causing light scattering and loss of transparency (Figure S19), rendering the constructs invisible under inverted optical microscopy (Figure 3e). We further exploited the changes in scattering by leveraging the photothermal activity of the organelle‐immobilized gold nanoparticles and monitored the processes using a thermal camera. NCVs without PNIPAAm exhibited pronounced photothermal transitions, heating from 24.2  to 67.4°C under near‐infrared irradiation (NIR, 808 nm, 6 W/cm^2^, 5 min). In contrast, PNIPAAm‐containing NCVs displayed self‐regulated responses with temperature increases slowed, reversed after ∼ 1 min, and then stabilized near the PNIPAAm LCST (31.5 –32.5°C) (Figures 3f,g and S20). This inhibition correlated with PNIPAAm concentration and was attributed to LCST‐driven aggregation (Figures S20 and S21). Furthermore, the NCVs displayed stable and reversible photothermal transitions under repeated light on–off cycles (Figure 3h). In these conditions, PNIPAAm‐containing artificial cells alternated between visible and invisible states depending on light exposure (Figure 3i), providing a mechanism to regulate light‐dependent metabolism‐like reactions within the artificial cells.

Based on these observations, free PNIPAAm was removed from the bulk solution by washing to produce a suspension of polymer‐loaded GOx/gold nanoparticle‐containing NCVs. Upon heating to 37°C, the gold nanoparticle‐loaded organelles became optically undetectable due to the formation of PNIPAAm aggregates inside the NCVs, while the overall NCV architecture remained visible because of the absence of external polymer aggregates (Figure S22). Although the interior PNIPAAm modestly influenced the photothermal behavior of the artificial cells, this effect was markedly less pronounced than when polymer was also present in bulk solution (Figure S23). However, the reversible formation of hydrophobic PNIPAAm domains under photothermal conditions enabled spatiotemporal cargo reorganization (e.g., dye molecules) within the NCVs by exchange between the NCV membrane and the internal aqueous microcompartment (Figure S24). Such reversible and spatially controlled rearrangements offer a promising strategy for achieving temperature‐ or light‐regulated (bio)chemical activity within confined, cell‐like architectures.

**FIGURE 4:**
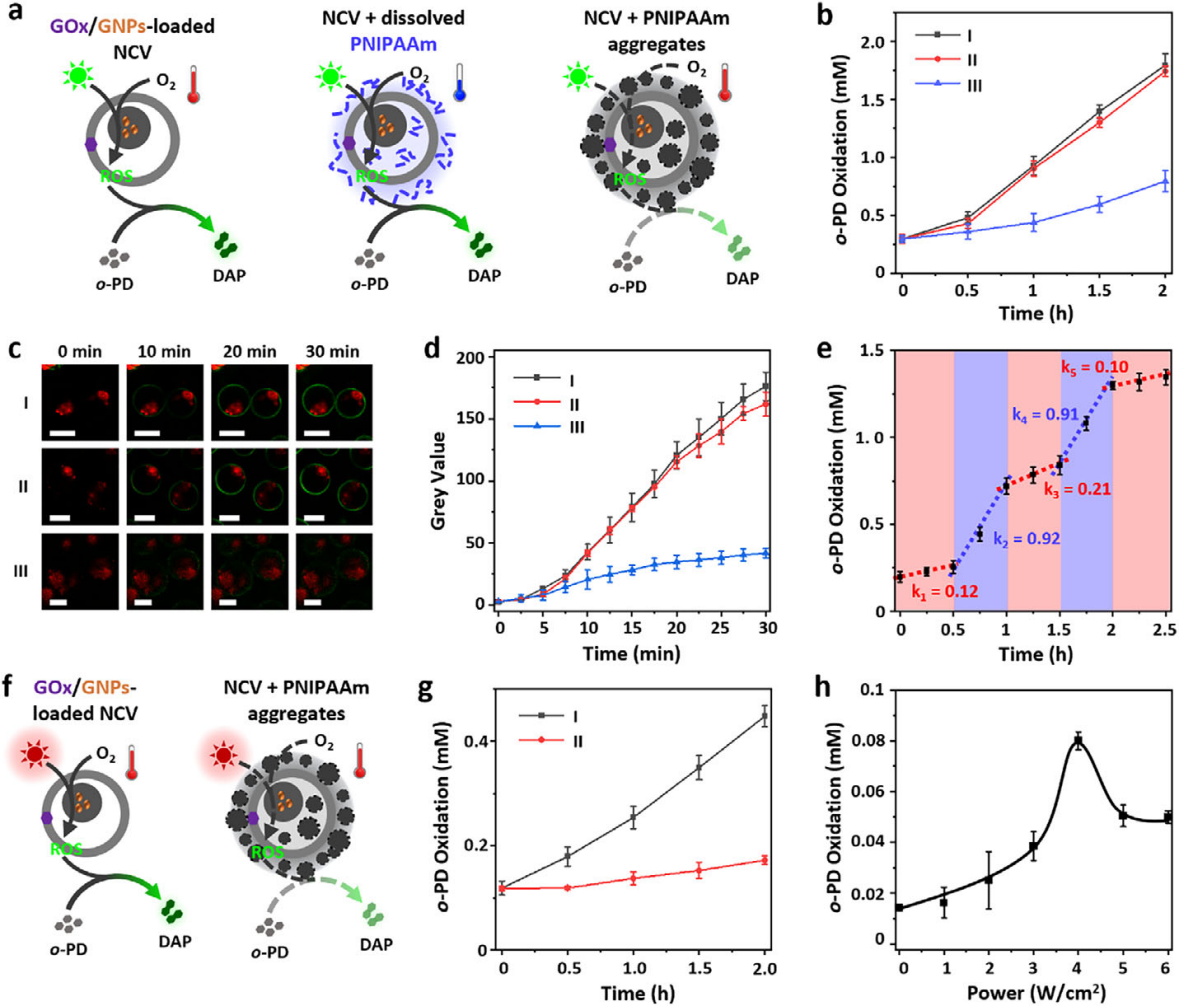
Dynamically regulated photocatalysis within NCVs. (a) Schematic illustrating how temperature‐dependent PNIPAAm aggregates regulate photocatalytic ROS generation and *o*‐phenylenediamine (*o*‐PD) oxidation to diaminophenazine (DAP) within NCV constructs under green light irradiation and at different temperatures. *o*‐PD oxidation is measured via the occurrence of UV absorbance (495 nm) and green fluorescence originating from DAP. (b) Plots illustrating *o*‐PD oxidation as a function of time in NCVs at different conditions. Group I: 34°C, without PNIPAAm; group II: 28°C, with self‐synthesized PNIPAAm; group III: 34°C, with self‐synthesized PNIPAAm. All samples were irradiated with green laser light (520 nm, ca. 0.1 W/cm^2^). (c) and (d) are corresponding time‐sequence CLSM images (c) and plots of green fluorescence intensity (gray values) (d) recording *o*‐PD oxidation in the three types of NCV in (b). The organelles are red fluorescent (RITC‐PAH). (e) *o*‐PD oxidation in PNIPAAm‐containing NCVs under cyclic temperature changes (34 ‐28 ‐34 ‐28 ‐34°C) with constant green light irradiation. (f) Schematic illustrating how temperature‐dependent PNIPAAm aggregates regulate photocatalytic ROS generation and *o*‐PD oxidation within NCV constructs under NIR light irradiation. (g) *o*‐PD oxidation in NCVs without (I) and with (II) self‐synthesized PNIPAAm under NIR irradiation (808 nm, ca. 6 W/cm^2^). (h) Plot recording *o*‐PD oxidation in PNIPAAm‐containing NCVs under NIR irradiation at varying laser intensities (0–6 W/cm^2^, ca. 1 h). Composition: PDDA/PAH/ATP, 20/10/12.5 mM; PTA, 3 mM; NIPAAm, 0 or 25 mg/mL. pH ca. 6.5. Error bars: *n* = 3 (repeated group of samples), mean value ± SD in (b,e,g,h); n ≥ 10, mean value ± SD in (d). Scale bars, 20 µm.

We next explored this regulatory feature by studying the photocatalytic behavior of the gold nanoparticle‐loaded organelles towards oxidation of *o*‐phenylenediamine (*o*‐PD) to diaminophenazine (DAP) within the PNIPAAm modified NCVs. Consistent with previous reports [41, 42], GOx/gold nanoparticle‐loaded NCVs without PNIPAAm converted dissolved O_2_ into reactive oxygen species (ROS) and catalysed *o*‐PD oxidation under green laser irradiation (520 nm, 100 mW/cm^2^) (Figure S25). Although DAP preferentially partitioned into the PAH/ATP guest domains, green fluorescence was also observed in the membrane‐like host domain, possibly arising from a reaction–diffusion process in which *o*‐PD and ROS diffuse toward the NCV membrane, where DAP forms in situ before redistributing. Reaction rates were modulated by O_2_ availability, temperature, and light exposure, with higher O_2_ levels and temperatures accelerating oxidation (Figures S25–S27). We investigated whether PNIPAAm‐mediated thermal regulation could invert this temperature dependence by preparing artificial cells with self‐synthesized PNIPAAm and incubating them with *o*‐PD (Figure 4a). Plots of DAP formation exhibited inverse behavior, with oxidation suppressed at higher temperature (0.25 mM/h at 34°C) and accelerated at lower temperature (0.72 mM/h at 28°C) (Figure 4b). The data were consistent with CLSM fluorescence readouts (Figure 4c,d). The change in temperature‐dependence was associated with PNIPAAm aggregation above the LCST (∼32°C), leading to light‐scattering and attenuated light penetration. As a result, PNIPAAm‐containing NCVs exhibited temperature‐dependent photocatalysis opposite to that of the PNIPAAm‐free controls (Figures 4e and S27). Similarly, NIR irradiation (808 nm), which induces photothermal heating, also drove *o*‐PD oxidation in the NCVs (Figure 4f). Without PNIPAAm, photocatalysis was efficient (0.16 mM h^−^^1^), whereas PNIPAAm‐containing NCVs showed markedly reduced activity (0.03 mM h^−^^1^) (Figure 4g), as photothermal heating triggered PNIPAAm‐induced light scattering and gave a nonlinear light‐intensity response with the rate of oxidation increased steadily from 1–4 W/cm^2^ but inhibited at ≥ 5 W/cm^2^, coinciding with PNIPAAm aggregation (Figure 4h).

## 3 Conclusion

In summary, we present a facile strategy to produce coacervate‐based membranous artificial cells with enclosed molecularly crowded sub‐compartments and self‐regulated catalytic activity. Precursor PDDA/PAH/ATP MCDs are reconfigured by the addition of PTA to produce a PDDA‐rich continuous membrane of complexed polyelectrolytes while preserving PAH/ATP coacervate guest domains, yielding reinforced NCV constructs. Unlike previous multiphasic systems prone to dissociation or coalescence and coacervate‐based vesicles lacking internal organization, the NCVs remain stable under variable environments, maintaining spatial segregation of their constituents and retaining catalytically active cargoes such as enzymes and gold nanoparticles. The reinforced architecture supports a quasi‐metabolic process by in situ synthesis of PNIPAAm, enabling the artificial cells to exhibit self‐regulated photothermal transitions and inverted, stimulus‐dependent photocatalysis. However, the negative feedback mechanism mediated by PNIPAAm aggregate‐induced light scattering lacks a significant time delay, causing the system to gradually reach a steady state rather than sustaining prolonged out‐of‐equilibrium dynamics. Moreover, the organelle‐like domains are highly restricted in their spatiotemporal reorganization and dynamic redistribution due to their mechanical robustness and physical anchoring to the membrane‐like domain of the NCVs.

Overall, this work provides an effective route to the construction of membranized and sub‐compartmentalized artificial cells capable of self‐sustained compositional and functional dynamics, offering new opportunities for the development of increasingly complex artificial cell systems. Future work will focus on developing new multiphasic coacervate systems and reconfiguration strategies to enhance matrix fluidity and dynamic behavior. Integrating the physicochemical properties of the coacervate matrix with embedded signaling cascades could enable time‐delayed feedback regulation, thereby promoting sustained out‐of‐equilibrium behaviors in nested artificial cells.

## Conflicts of Interest

The authors declare no conflicts of interest.

## Supporting information




**Supporting File**: anie72104‐sup‐0001‐SuppMat.docx.

## Data Availability

The data that support the findings of this study are available in the Supporting Information of this article.
