# Supplementary material for: Reconfiguration of Multiphase Coacervate Droplets Into Self‐Regulated Nested Artificial Cells
Source: Angew Chem Int Ed Engl. 2026 Apr 10;65(21):e9334343. doi: 10.1002/anie.9334343 (PMC13182209; doi:10.1002/anie.9334343)
Supplement: Supplementary file 1 — Supporting File: anie72104‐sup‐0001‐SuppMat.docx. [file ANIE-65-e9334343-s001.docx]

**Reconfiguration of multiphase coacervate droplets into self-regulated nested artificial cells**

Zhuping Yin, ^[a] [b]^ Rui Sun, ^[a]^ Jingxin Shao, ^[b]^ Jan C.M. van Hest*^[b]^ and Stephen Mann*^[a] [c]^

[a] Dr. Z. Yin, Dr. R. Sun, Prof. S. Mann
Centre for Protolife Research and Centre for Organized Matter Chemistry, School of Chemistry, University of Bristol, Bristol BS8 1TS, United Kingdom.

Email: s.mann@bristol.ac.uk

[b] Dr. Z. Yin, Dr. J. Shao, Prof. J. C.M. van Hest
Bio-Organic Chemistry, Departments of Biomedical Engineering and Chemical Engineering & Chemistry, Institute for Complex Molecular Systems, Eindhoven University of Technology, 5600 MB Eindhoven, The Netherlands.

Email: J.C.M.v.Hest@tue.nl

[c] Prof. S. Mann
Max Planck-Bristol Centre for Minimal Biology, School of Chemistry, University of Bristol, Bristol BS8 1TS, United Kingdom.

**Supplementary Methods and Supplementary Figures**

**Contents**

[**1. Supplementary Materials and Methods** 3](#_Toc224569214)

[**1.1 Materials** 3](#_Toc224569215)

[**1.2 Synthesis of poly(diallyl dimethylammonium chloride)-co-diallylamine-rhodamine (RITC-PDDA)** 3](#_Toc224569216)

[**1.3 Preparation of FITC-labelled components** 3](#_Toc224569217)

[**1.4 Preparation and characterization of multiphase coacervate droplets and nested coacervate vesicles** 4](#_Toc224569218)

[**1.5 Cargo loading within multiphase coacervate droplets and nested coacervate vesicles** 5](#_Toc224569219)

[**1.6 GOx/Gold nanoparticle (GNP) -mediated synthesis of PNIPAAm in H_2_O** 6](#_Toc224569220)

[**1.7 Synthesis of PNIPAAm within GOx/GNP-loaded coacervate systems** 6](#_Toc224569221)

[**1.8 Photothermal effect of GOx/GNP-loaded nested coacervate vesicles** 7](#_Toc224569222)

[**1.9 Photocatalytic behaviour of GOx/GNP-loaded nested coacervate vesicles** 7](#_Toc224569223)

[**1.10 Photon-induced spatial confinement of cargoes within GOx/GNP-loaded nested coacervate vesicles** 8](#_Toc224569224)

[**2. References** 8](#_Toc224569225)

[**3. Supplementary Figures** 9](#_Toc224569226)

# **1. Supplementary Materials and Methods**

## **1.1 Materials**

The following chemicals were used as received: poly(diallyl dimethylammonium chloride) solution (PDDA, Mw: 100-200 kD, Sigma-Aldrich), polyallylamine hydrochloride (PAH, Mw: 50 kD, Sigma-Aldrich), FITC-labelled CM-dextran (Sigma-Aldrich, Mw: 70 kD), FITC-labelled DEAE-dextran (Sigma-Aldrich, Mw: 70 kD), FITC-labelled dextran (Sigma-Aldrich, Mw: 4-250 kD), α-amylase from porcine pancreas (Type I-A, ≥1000 units/mg protein, Sigma-Aldrich), glucose oxidase from *Aspergillus niger* (GOx, Type X-S, lyophilized powder, 100,000-250,000 units/g solid, Sigma-Aldrich), peroxidase from horseradish (HRP, ~ 150 units/mg protein, Sigma-Aldrich), adenosine 5′-triphosphate disodium salt hydrate (ATP, Sigma-Aldrich), sodium phosphotungstate hydrate (PTA, Sigma-Aldrich), fluorescein isothiocyanate isomer I (FITC, ≥ 90%, Sigma-Aldrich), rhodamine B isothiocyanate (RITC, Sigma-Aldrich), gold nanoparticles (GNP, size: 20 nm, Sigma-Aldrich), HS-PEG-NH_2_ (Mw: 2 kD, Sigma-Aldrich), D-(+)-glucose (≥ 99.5%, Sigma-Aldrich), *N*-isopropylacrylamide (NIPAAm, Sigma-Aldrich, Mw: 113.16), diallylamine (≥ 99.0%, Sigma-Aldrich), 2,2'-azobis(2-methylpropionamidine)dihydrochloride (AAPH, ≥ 97.0%, Sigma-Aldrich), diallyldimethylammonium chloride solution (DADMAC, 65% in H_2_O, Sigma-Aldrich), *o*-phenylenediamine (*o*-PD, ≥ 99.0%, Mw: 108.14, Sigma-Aldrich).

## **1.2 Synthesis of poly(diallyl dimethylammonium chloride)-co-diallylamine-rhodamine (RITC-PDDA)**

Diallylamine (97.2 mg, *ca.* 1 mmol) and AAPH initiator (30 mg) were dissolved in H_2_O (2 ml) and added to diallyl dimethylammonium chloride solution (DADMAC, 24.872 g, *ca.* 100 mmol, 65 wt%), followed by setting the whole volume to 100 ml using H_2_O. The reaction was started by adjusting the pH value to *ca.* 3.0 and then heating to 60 ^o^C, followed by stirring for 24 h**^1^**. The product was fluorescently labelled by adding RITC (2 mg/ml, DMSO, 1 ml) and setting the pH value to ca. 8.5 using carbonate buffer (Na_2_CO_3_/NaHCO_3_, 100/100 mM). The reaction mixture was stirred for over 8 h and the RITC-labelled product was then purified by dialysis against DI water using a cellulose membrane (MWCO, 12-14 kD) and lyophilization. The collected RITC-PDDA was then dissolved in non-fluorescent PDDA solution (Mw, 100-200 kD; concentration, 200 mM) in the ratio of 1/200 (w/w) and stored at 4 ^o^C for usage.

## **1.3 Preparation of FITC-labelled components**

**FITC-labelled PAH:** Poly(allylamine hydrochloride) (PAH, Mw: *ca.* 50 kDa, 100 mg) was dissolved in H_2_O (50 ml), and the pH was adjusted to *ca.* 4.5 with NaOH (0.5 M). FITC (100 μl, 2 mg/mL in DMSO) was added, and the solution was stirred at R.T. for *ca.* 12 h. The product was purified by dialysis against DI water at pH 4 (MWCO 12-14 kDa, 3-4 days) and lyophilized.

**FITC-labelled enzymes:** α-Amylase, glucose oxidase (GOx), and horseradish peroxidase (HRP) (10 mg each) were dissolved in carbonate buffer (Na_2_CO_3_/NaHCO_3_, 25/25 mM, pH 8.5, 10 ml). FITC (50 μl, 2 mg/ml in DMSO) was added, and the mixtures were stirred at R.T. for ca. 12 h. The products were purified by dialysis against DI water (MWCO 12-14 kDa, 3-4 days) and lyophilized.

**FITC-labelled gold nanoparticles:** Commercial citrate-stabilized gold nanoparticles (20 nm, OD_520_ = *ca.* 1) were mixed with FITC–PEG–SH (Mw, 2 kDa; 20–50 μM final; ≥10^4^ ligands/particle) and stirred at R.T. for 2 h, followed by stirring at R.T. overnight^2^. The suspension was then stirred in 10 mM NaCl solution over 60 min. Particles were purified from excess ligand by repeated centrifugation (10,000 rpm, 15 min) and redispersion into HEPES buffer (10 mM, 10 mM NaCl, pH 7.4). Final suspensions were adjusted to OD_520_ = *ca.* 1 and stored at 4 ^o^C in the dark.

## **1.4 Preparation and characterization of multiphase coacervate droplets and nested coacervate vesicles**

**Formation of multiphase coacervate droplets and nested coacervate vesicles:** A range of multiphasic complex coacervate droplets with different morphologies were prepared by altering ATP concentrations (10-30 mM) and pH values (3-9) while keeping PDDA (20 mM) and PAH (10 mM) constant. Typically, ATP solution (100 mM, pH *ca.* 4, 20-60 µl) was added to mixtures of PDDA (200 mM, 20 µl, pH *ca.* 4) and PAH (100 mM, 20 µl, pH *ca.* 4), and the total volume was adjusted to 200 µl with DI water. pH was tuned to 3.0-9.0 by stoichiometric addition of NaOH (0.5 M) or HCl (0.5 M). Dispersions were transferred to a sealed chamber (Scheme 1) and incubated at R.T. for 12-24 h. RITC-labelled PDDA (RITC-PDDA/PDDA, 1/200 (m/m), 200 mM), FITC-labelled PAH (100 mM, pH *ca.* 4) and TNP-ATP (TNP-ATP/ATP, 1/100 (n/n), 100 mM, pH *ca.* 4.0) were introduced in these processes to observe compositional organization within the generated coacervate droplets.


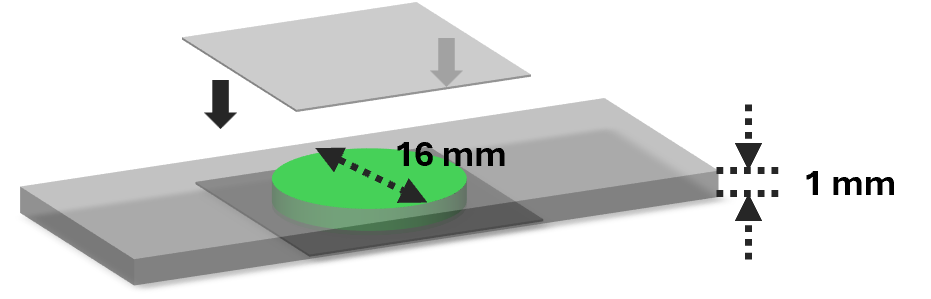


**Supplementary scheme 1** A glass slide with a circular chamber (green, diameter: 16 mm, thickness: 1 mm) sealed between two cover slips using glue. The aqueous sample was introduced into the chamber prior to sealing with the top cover slip.

Nested coacervate vesicles were produced by inducing *in situ* morphological reconfiguration of multiphasic coacervate droplets with sodium phosphotungstate hydrate (PTA, *ca.* 3 mM)^3^. After static incubation of the multiphasic droplets (200 µl), PTA solution (100 mM, 6 µl, pH 3-9) was added dropwise, gently mixed by pipetting, and incubated at R.T. for 15 min. pH values were recorded using a pH meter (Mettler Toledo, Switzerland). Bright-field and fluorescence microscopy (Leica DMI3000 B) and confocal laser scanning microscopy (CLSM, SP5-II, Leica) were used to monitor coacervate formation, reconfiguration, and compositional distribution. Images were analysed with Image J software. After removal of supernatant and lyophilization, SEM and SEM-EDS mapping were used to examine the morphologies and elemental distributions (P, W) of the lyophilized microstructures. Zeta potentials (ζ) of dispersions were measured on a Zetasizer Nano ZSP (Malvern, UK).

**Matrix viscoelasticity and compositional association strength:** Fluorescence recovery after photobleaching (FRAP) was performed by CLSM to characterize matrix viscoelasticity of multiphasic coacervate droplets (PDDA/PAH/ATP, 20/10/12.5 mM) and nested vesicles (PDDA/PAH/ATP/PTA, 20/10/12.5/3 mM), prepared at pH *ca.* 4 and 6.5. Guest and host domains of the coacervate microstructures were separately selected for FRAP analysis. The mobile fraction was calculated according to:

$$\text{Mobile fraction}=\frac{F_{\infty}-F_{0}}{F_{i}-F_{0}}$$

where $F_{i}$is the fluorescence intensity before photobleaching, $F_{0}$is the fluorescence intensity immediately after bleaching, and $F_{\infty}$is the fluorescence intensity after recovery reached a plateau. The immobile fraction was determined as:

$$\text{Immobile fraction}=1-\text{Mobile fraction}$$

The resulting recovery curves and calculated fractions were used to compare the viscoelastic properties of the host and guest domains in the MCDs and NCVs.

Morphological stability was tested by adding NaCl solution (0-20 µl, 2 M) to dispersions of multiphasic coacervate droplets and nested coacervate vesicles and incubating at R.T. for 24 h, followed by imaging using CLSM and analysis using Image J software.

**Permeability of nested coacervate vesicles:** Dispersions of nested vesicles (200 µl) were prepared at PDDA/RITC-PAH/ATP/PTA concentrations of 20/10/12.5/3 mM and pH *ca.* 6.5 as described above, then supplemented with FITC-dextran (Mw 4-250 kDa, 0.05 mg/ml, 4 µl) and incubated at R.T. for 0-24 h. Time-dependent CLSM was used to monitor FITC-dextran distribution. Partition coefficients were calculated as the ratio of fluorescence intensity (grey value) inside vs. outside vesicles using Image J software.

## **1.5 Cargo loading within multiphase coacervate droplets and nested coacervate vesicles**

**Fluorescence dye loading:** Rhodamine 123, sulforhodamine B, and Nile red (1 mg each) were dissolved in 1 mL of H_2_O (rhodamine 123 and sulforhodamine B) or DMSO (Nile red) and subsequently diluted 20-fold with deionized water. Aliquots (4 μL, 0.05 mg/ml) of each dye solution were added separately to aqueous dispersions of multiphase coacervate droplets (200 μL) prepared from PDDA/PAH/ATP at concentrations of 20/10/12.5 mM (pH 4.0). The pH was then adjusted to *ca.* 6.5 by stoichiometric addition of NaOH (0.5 M, *ca.* 4 μL), followed by quiescent incubation at room temperature for 2 h. PTA (100 mM, 6 μL) was added to the dye-loaded droplets to induce reconfiguration into nested coacervate vesicles. Fluorescence distributions were characterized by CLSM and analysed using ImageJ software.

**FITC-polysaccharide loading:** FITC-labeled CM-dextran (Mw = 40 kDa) and DEAE-dextran (0.2 mg/ml, 5 μL each) were separately added to dispersions of multiphase coacervate droplets (200 μL; PDDA/PAH/ATP, 20/10/12.5 mM; pH 4.0). The pH was adjusted to *ca.* 6.5, and the samples were incubated at room temperature for 2 h before inducing reconfiguration into nested coacervate vesicles by addition of PTA (100 mM, 6 μL). Fluorescence distributions were characterized by CLSM and analysed using ImageJ software.

**FITC-enzyme loading:** FITC-labelled α-amylase, GOx, and HRP (1 mg/ml, 10 μL) were separately added to dispersions of multiphase coacervate droplets (200 μL; PDDA/PAH/ATP, 20/10/12.5 mM; pH 4.0). The pH was adjusted to ca. 6.5, and the samples were incubated at room temperature for 2 h. Nested coacervate vesicles were generated by addition of PTA (100 mM, 6 μL).

The spatial distribution of FITC-GOx within multiphase coacervate droplets and nested vesicles was evaluated under saline (NaCl, 100 mM) and proteolytic (protease XIV from *Streptomyces griseus*, 3.5 unit/ml) conditions. Typically, dispersions of FITC-GOx-loaded droplets or vesicles (200 μL) were prepared as described above, followed by addition of NaCl (0-20 μL, 1 M) or protease XIV (35 unit/ml). The FITC-GOx-loaded dispersions were then incubated at R.T. for 0-48 h, observed by CLSM and analysed using Image J software.

The sustained bioactivity of the GOx/HRP enzyme cascade was evaluated under proteolytic conditions. Typically, dispersions of GOx/HRP-loaded multiphasic coacervate droplets and nested coacervate vesicles were prepared as described above, followed by the addition of protease XIV (0 or 35 U mL⁻¹) and quiescent incubation for 24 h. Afterwards, enzyme cascade activity was evaluated by monitoring o-PD oxidation (20 mM) in the coacervate systems containing glucose (10 mM) by recording UV–vis absorption spectra (350–650 nm).

**Gold nanoparticle-loading:** FITC-PEG-SH-coated gold nanoparticles (FITC-GNPs, 20 nm, OD_520_ = *ca.* 1, 10 µl) were mixed with PDDA (200 mM, 20 µl, pH 4) and PAH (100 mM, pH 4, 20 µl), followed by addition of ATP (100 mM, pH 4, 25 µl) to induce coacervation and setting the whole volume to 200 µl with H_2_O. The suspension was incubated at R.T. for *ca.* 24 h, followed by setting the pH to *ca.* 6.5 and further incubating the suspension for 2 hours without agitation. PTA (100 mM, pH 6.5, 6 µl) was added to produce nested coacervate vesicles. The distribution of FITC-GNPs was observed by CLSM and analysed using Image J software.

In supplementary experiments, non-fluorescent gold nanoparticles (20 nm, OD₅₂₀ ≈ 4, 50 µL) were mixed with PDDA (200 mM, 20 µL, pH 4) and PAH (100 mM, 20 µL, pH 4), followed by the addition of ATP (100 mM, pH 4, 25 µL) to induce coacervation, and the total volume was adjusted to 200 µL with H₂O. The suspension was then modified in pH value ca. 6.5, followed by adding PTA (100 mM, pH 6.5, 0 or 6 µL) to produce nested coacervate vesicles and an incubation for 1-7 days. Afterwards, the aggregation state of the encapsulated gold nanoparticles was evaluated by recording UV–vis absorption spectra after releasing the nanoparticles by adding NaCl (5 M, 400 µL) and HCl (5 M, 200 µL) under vortex mixing (1000 rpm, 15 s) and rapidly adjusting the pH values to 6.5 prior to measurement.

## **1.6 GOx/Gold nanoparticle (GNP) -mediated synthesis of PNIPAAm in H_2_O**

A reaction cascade of glucose oxidase (GOx) and GNPs was employed to generate free radicals and induce polymerization of *N*-isopropylacrylamide (NIPAAm) under aqueous conditions with glucose and low O_2_ levels. NIPAAm monomer (50 mg) was dissolved in D_2_O (250 μl) and added to a mixture containing GOx (1 mg/mL in D_2_O, 200 μl), commercial GNPs (20 nm citrate-stabilized, OD_520_ = *ca.* 1, 1000 μl), and PBS buffer (pH 7.0, 10 mg/mL in D_2_O, 500 μl). Glucose (500 mM in D_2_O, 40 μl) was then added, and the total volume was adjusted to 2000 μl with D_2_O. The mixture was sealed in a 7 mL glass vial with a rubber septum, flushed with N_2_ for 15 min to remove headspace O_2_, and incubated at R.T. under stirring for 0–24 h. Polymerization was quenched by exposure to air. Products were analysed by ¹H NMR spectroscopy (400 MHz, Bruker) using the HDO resonance as internal standard. Proton numbers (nH) were determined by spectral integration. Reaction mixtures collected at 24 h were also photographed at 25 and 37 ^o^C.

## **1.7 Synthesis of PNIPAAm within GOx/GNP-loaded coacervate systems**

GOx and GNPs were spatially confined within multiphasic coacervate droplets and nested coacervate vesicles to enable *in-situ* synthesis of PNIPAAm. Dispersions of GOx/GNP-loaded multiphase coacervate droplets were prepared by mixing GOx (2 mg/ml, 5 µl) and GNPs (20 nm citrate-stabilized, OD₅₂₀ = *ca.* 4, 50 µl) with PDDA (200 mM, pH 4, 20 µl), PAH (100 mM, pH 4, 20 µl) and NIPAAm monomer (250 mg/ml, 20 µl), inducing complex coacervation by adding ATP (100 mM, pH 4, 25 µl) and setting the whole volume to 200 µl by adding H_2_O. The mixtures were transferred to glass-based chambers and incubated for *ca.* 24 h, followed by adjusting the pH value to *ca.* 6.5 and inducing reconfiguration into nested coacervate vesicles by adding PTA (100 mM, 0 or 6 µl). The polymerization of NIPAAm was implemented by adding glucose (500 mM, 6 µl), sealing the chambers using coverslips, and quiescent incubation at R.T. for 24 h. Time-dependent images were collected by inverted optical microscopy to monitor polymerization at 25 and 37 ^o^C.

PNIPAAm formed in the background phase was isolated by centrifuging (10,000 rpm, 10 min, R.T.) the nested coacervate vesicle dispersion, collecting the supernatant, followed by purification via repeated centrifugation at 37 °C (10,000 rpm, 10 min) and redissolution at 4 °C (×3). The purified PNIPAAm was lyophilized, redissolved in THF (1 mg/mL), and analysed by MALDI-ToF mass spectrometry (Bruker, Germany).

## **1.8 Photothermal effect of GOx/GNP-loaded nested coacervate vesicles**

Dispersions of GOx/GNP-loaded nested coacervate vesicles containing varying levels of PNIPAAm were generated as described above by inducing *N*-isopropylacrylamide (NIPAAm) polymerization at 0, 25, or 50 mg/ml. Dispersions were transferred into 1.5 mL Eppendorf tubes and subjected to photothermal activation by exposure to a near-infrared laser (808 nm, MDL-III-808, 0-6 W/cm²). The transition was repeatedly triggered and quenched by ON-OFF cycling of the light source. Temperature changes were monitored using a thermal camera (FLIR, Estonia) and analysed with FLIR software. Inverted optical microscopy was employed to observe the nested coacervate vesicles sealed in a glass chamber during photothermal transitions.

## **1.9 Photocatalytic behaviour of GOx/GNP-loaded nested coacervate vesicles**

**Without PNIPAAm:** Dispersions of GOx/GNP-loaded nested vesicles (PDDA/PAH/ATP/PTA, 20/10/12.5/3 mM; pH *ca.* 6.5; 200 µl) were prepared without NIPAAm polymerization and transferred into 1.5 ml Eppendorf tubes. After adding *o*-phenylenediamine (*o*-PD, 200 mM, pH *ca.* 6.5, 10 µl) and glucose (500 mM, 0 or 4 μL), oxidation of *o*-PD was triggered by irradiation with a green laser (0 or 0.1 W/cm^2^, 0-2 h). Reaction rates were modulated by N_2_ flushing to remove dissolved O_2_ or by varying the temperature (25-45 ^o^C).

*o*-PD oxidation was quantitively defined by UV/Vis spectroscopy (Perkin Elmer Lambda 750) via absorbance of 2,3-diaminophenazine (DAP) at 495 nm. At designated time points (every 0.5 h), 20 µl aliquots were withdrawn, mixed with HCl (5 M, 200 µl) and H₂O (780 µl), vortexed (1000 rpm, 15 s), and centrifuged (10,000 rpm, 5 min) to remove aggregates. Supernatants were measured at 495 nm, and standard plots obtained from known concentrations of DAP (0-0.01 mM) were used to calibrate the reaction. Photocatalytic o-PD oxidation was also examined in a glass chamber containing the nested coacervate vesicles and monitored by CLSM (excitation 488 nm, emission 498-550 nm) through the appearance of DAP fluorescence. Images were analysed using ImageJ software.

**With PNIPAAm:** Dispersions of GOx/GNP-loaded nested coacervate vesicles were prepared as above (PDDA/PAH/ATP/PTA, 20/10/12.5/3 mM; NIPAAm, 25 mg/ml; pH *ca.* 6.5; 200 µl). PNIPAAm was synthesized *in situ* by adding glucose (500 mM, 6 µl) and incubating at R.T. for 24 h.

The dispersions were transferred into 1.5 mL Eppendorf tubes and mixed with *o*-PD (200 mM, pH 6.5, 10 µl). *o*-PD oxidation was initiated by green laser irradiation (0.1 W/cm^2^) and kinetically modulated by adjusting the reaction temperature (28 or 34 ^o^C). The absorbance of DAP at 495 nm was recorded every 30 min as described above. Dynamic modulation was further examined by maintaining the light source (0.1 W/cm^2^) while alternating the reaction temperature between 28 and 34 °C every 0.5 h, with oxidation levels quantified every 15 min. Additionally, near-infrared (NIR) irradiation (808 nm, 0–6 W/cm^2^) was applied to trigger o-PD oxidation within the nested coacervate vesicles, and absorbance was recorded as described above.

Supplementarily, photocatalytic o-PD oxidation was also examined by incubating the nested coacervate vesicles in a sealed glass chamber and monitoring the appearance of DAP fluorescence using CLSM (excitation 488 nm, emission 498-550 nm). Images were analysed with ImageJ software.

## **1.10 Photon-induced spatial confinement of cargoes within GOx/GNP-loaded nested coacervate vesicles**

Resorufin, rhodamine 123, rhodamine 6G, and Nile red (1 mg each) were separately dissolved in DMSO (1 mL) and diluted 20-fold with deionized (DI) water. Dispersions of GOx/GNP-loaded nested coacervate vesicles containing PNIPAAm were prepared as described above (PDDA/PAH/ATP/PTA, 20/10/12.5/3 mM; NIPAAm, 25 mg mL⁻¹; pH *ca.* 6.5; 200 µL). PNIPAAm was synthesized *in situ* by adding glucose (500 mM, 6 µL), followed by incubation at R.T for 24 h. The dispersions were transferred into 1.5 mL Eppendorf tubes and centrifuged (1000 rpm, 3 min). Supernatants were discarded and replaced with DI water (ca. 200 µL) to remove background compounds. The purified dispersions were then mixed with 10 µL of dye stock solutions (resorufin, rhodamine 123, rhodamine 6G, or Nile red; 0.05 mg/ml) and sealed in a glass-based chamber. Samples were incubated quiescently at RT for 2 h.

Fluorescence distribution within the nested coacervate vesicles was regulated by cyclic near-infrared (NIR) laser irradiation (808 nm, 0 or 6 W/cm^2^, 2 min) and observed by time-dependent CLSM imaging. Images were then analysed using ImageJ software.

# **2. References**

1. Moreau, N. G.; Martin, N.; Gobbo, P.; Tang, T. Y. D.; Mann, S. Spontaneous Membrane-Less Multi-Compartmentalization via Aqueous Two-Phase Separation in Complex Coacervate Microdroplets. *Chem. Commun.* 2020, 56, 12717–12720.
2. Wang, J.; Xin, Y.; Chen, D.; Zhang, N.; Xue, Y.; Liu, X.; Li, X.; Gao, W.; Hu, Z.; Sun, T.; Liu, K. Ultra-Stable Gold Nanoparticles with Tunable Surface Characteristics. *Angew. Chem. Int. Ed.* 2025, e202507954.
3. Williams, D. S.; Patil, A. J.; Mann, S. Spontaneous Structuration in Coacervate-Based Protocells. *Small* 2014, 10, 1830–1840.

# **3. Supplementary Figures**


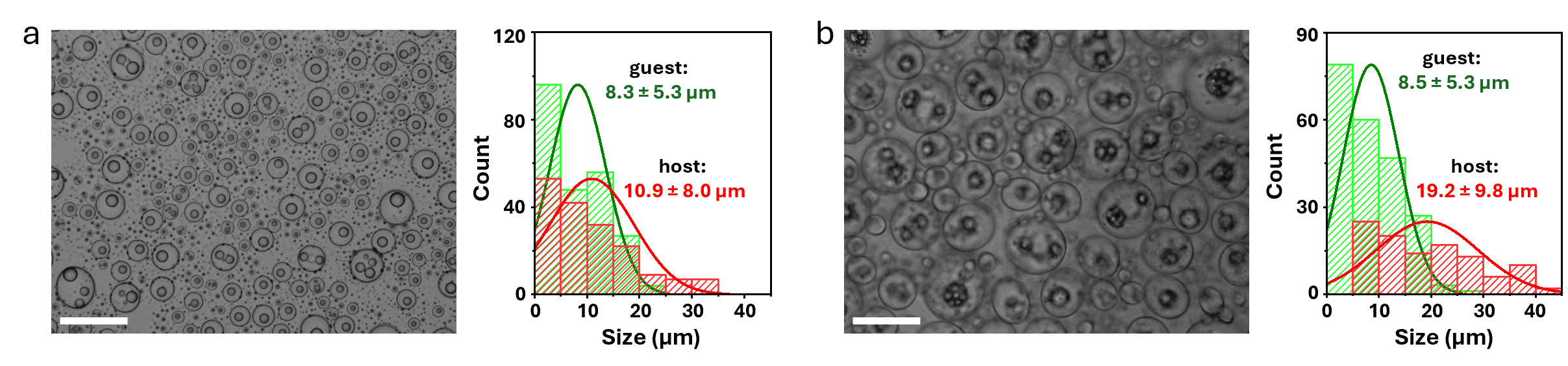


**Supplementary Figure 1.** (a) and (b) are bright field microscopic images (left) and size distributions (right) of MCD and NCV constructs, respectively. Guest (green) and host (red) entities are analysed separately in the size distribution using Image J software. Composition: PDDA/ PAH/ATP, 20/10/12.5 mM; PTA, 3 mM. The MCD was formed at pH 4.5 and statically incubated at R.T. for 12 h. NCV was then formed by addition of PTA (3 mM, pH 7). Scale bars, 50 μm.


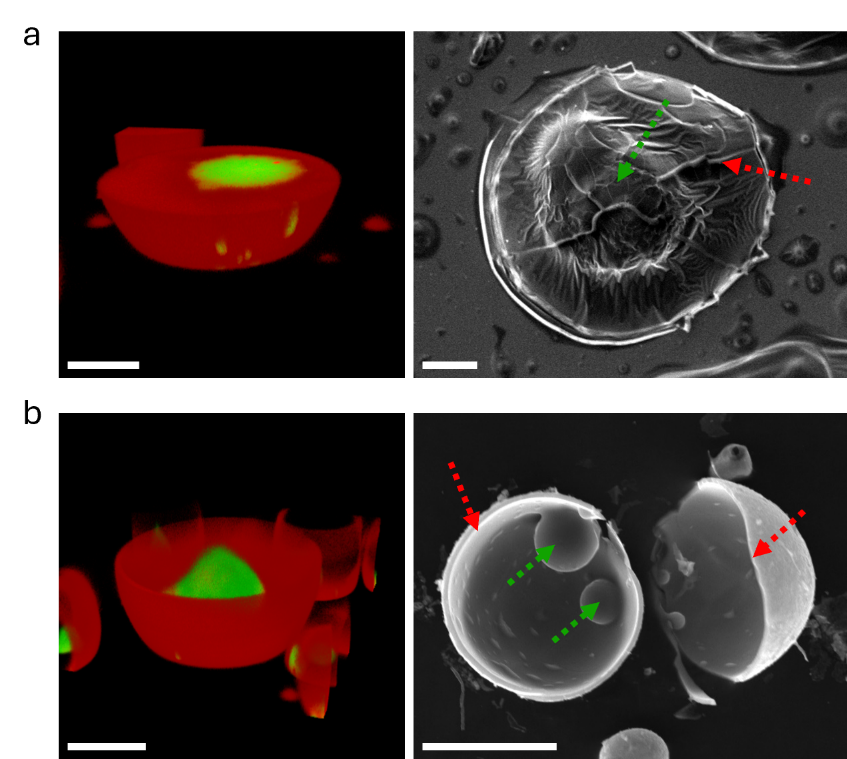


**Supplementary Figure 2**. (**a**) and (**b**) are MCD and NCV constructs, respectively, recorded by 3D stacked CLSM (left) and SEM imaging (right). Composition: RITC-labelled PDDA (red)/FITC-labelled PAH (green)/non-fluorescent ATP, 20/10/12.5 mM; PTA, 3 mM. The MCD was formed at pH 4.5 and incubated at R.T. for 12 h. The generated dispersions of MCD and NCV constructs were lyophilized and then imaged by SEM. Green dashed arrows indicate the sites of inner PAH droplets while the red dashed arrows point out the outer coacervate phase. Scale bars, 20 μm.

**
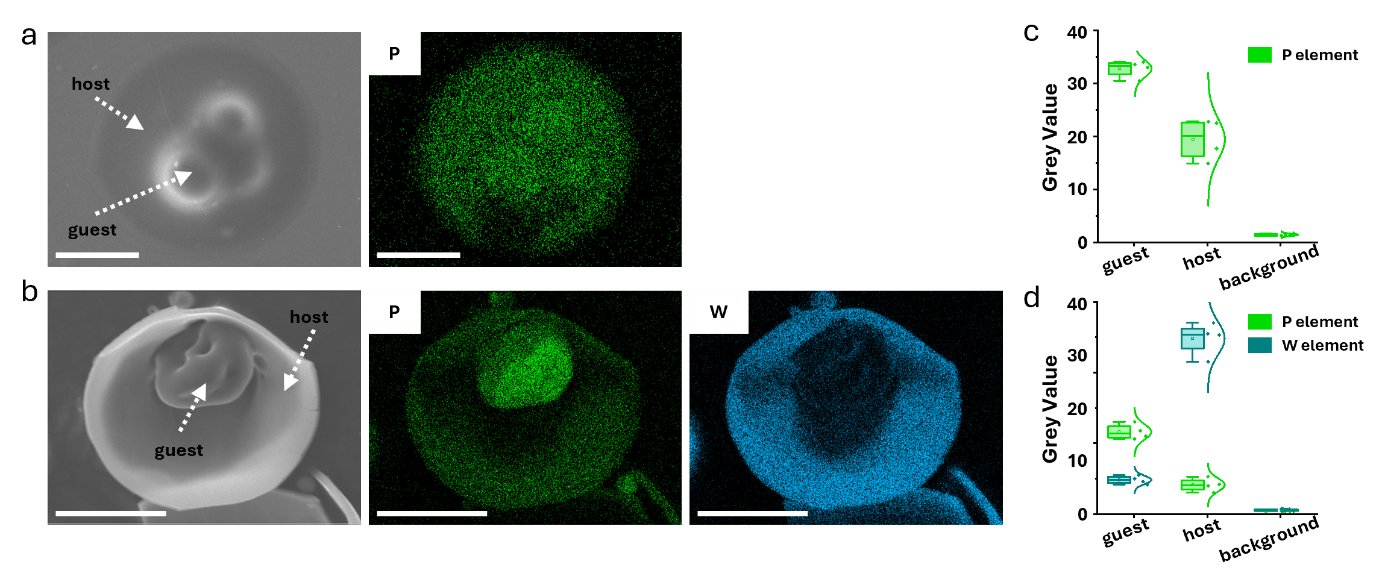
**

**Supplementary Figure 3.** SEM-EDS elemental mapping showing site-specific distribution of phosphorus and tungsten in MCD (**a**) and NCV (**b**). (**c**) and (**d**) are corresponding quantitative analysis of the phosphorus and tungsten distributions obtained by measuring site-specific grey values using ImageJ software. Phosphorus is more enriched within the inner droplets of MCD and NCV. Tungsten is mostly distributed within the host coacervate vesicle of NCV. Composition: PDDA/PAH/ATP, 20/10/12.5 mM; PTA, 0 (a) and 3 mM (b). Error bars: n = 4, mean value ± SD. Scale bars, 20 μm.


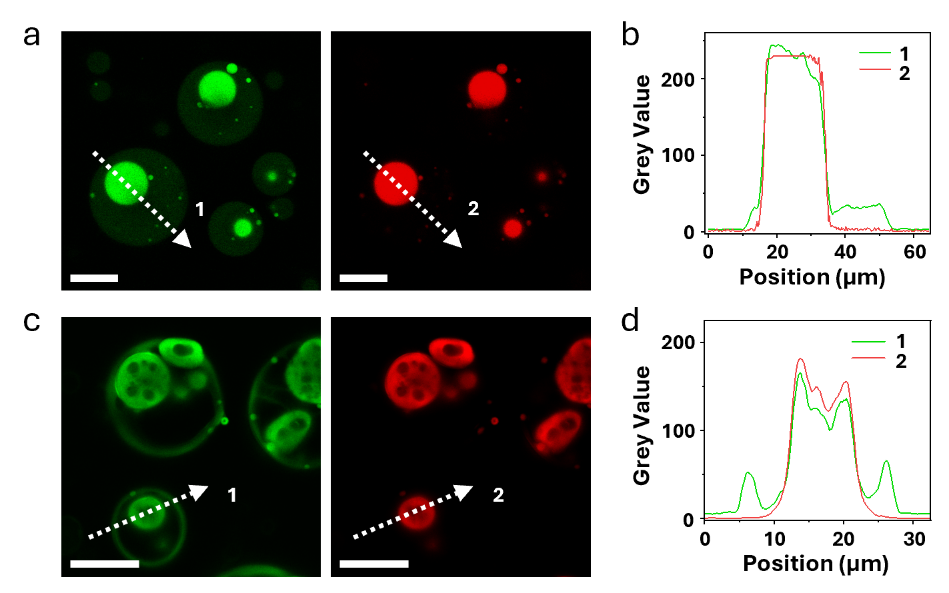


**Supplementary Figure 4.** (**a**) and (**c**) are CLSM images of MCD and NCV constructs, respectively. Composition: PDDA/RITC-PAH/TNP-ATP, 20/10/12.5 mM; PTA, 0 (**a**) or 3 mM (**c**). RITC-PAH is red and TNP-ATP is green fluorescent. (**b**) and (**d**) are line profiles indicating the distribution of TNP-ATP (1) and RITC-PAH (2) in MCD (**a**) and NCV (**c**). Scale bars, 20 µm.


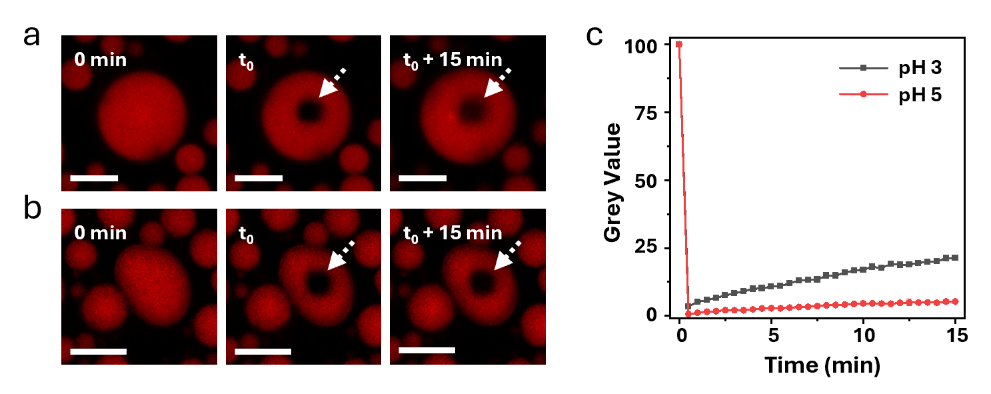


**Supplementary Figure 5.** (**a**) and (**b**) are CLSM images showing the FRAP process of PAH/ATP coacervates prepared at pH 3 (**a**) and 5 (**b**) respectively. Composition: RITC-PAH/TNP-ATP, 20/10 mM. White arrows highlight the bleached areas within the coacervate phase. (**c**) presents the FRAP profiles of the coacervate systems in (**a**) and (**b**). Red signal originates from RITC-PAH. Scale bars, 10 µm.


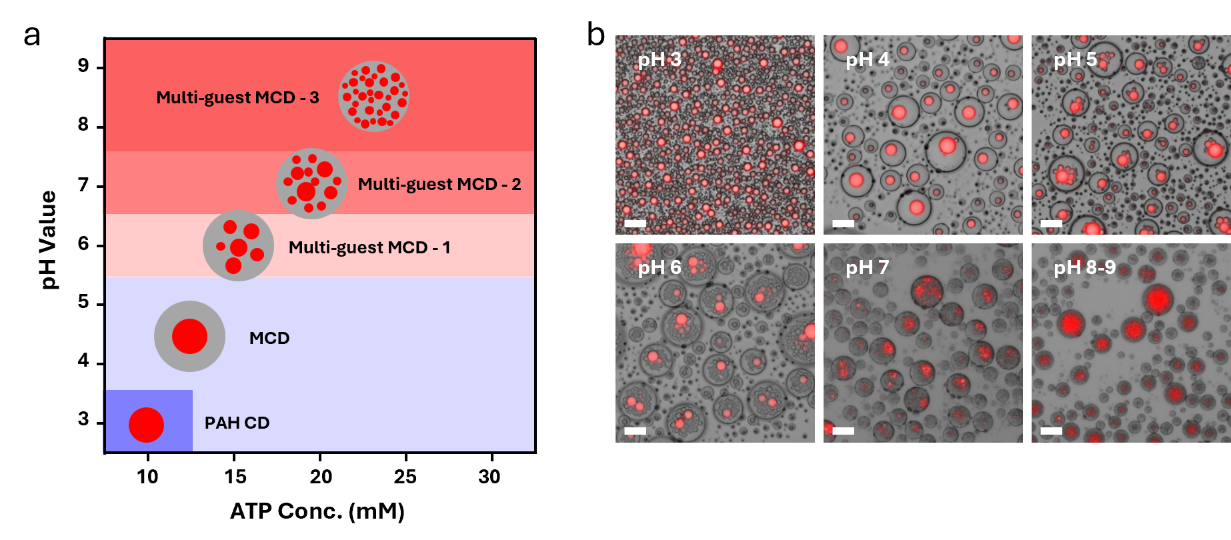


**Supplementary Figure 6.** (**a**) State diagram showing morphologies of coacervate droplets formed under different conditions. Composition: PDDA, 20 mM; PAH, 10 mM; ATP, 10-30 mM; pH 3-9. (**b**) Representative images of PDDA/PAH/ATP droplets prepared at identical ATP concentration (10 mM) and varying pH. PAH was RITC-labeled. Altering pH from 3 to 9 produced distinct morphologies: PAH CD (PAH/ATP, pH 3; size 2-10 μm); MCD (PDDA/PAH/ATP, pH 4-5; host 3-30 μm, guest 2-20 μm, 1-2 guests/host); multi-guested MCD-1 (pH 6; host 3-30 μm, guest 2-5 μm, 5-50 guests/host); multi-guested MCD-2 (pH 7; host 3-30 μm, guest 0.5-3 μm, >100 guests/host); multi-guested MCD-3 (pH 8-9; host 3-30 μm, guest 0.5-1 μm, >100 guests/host). Scale bars, 20 μm.


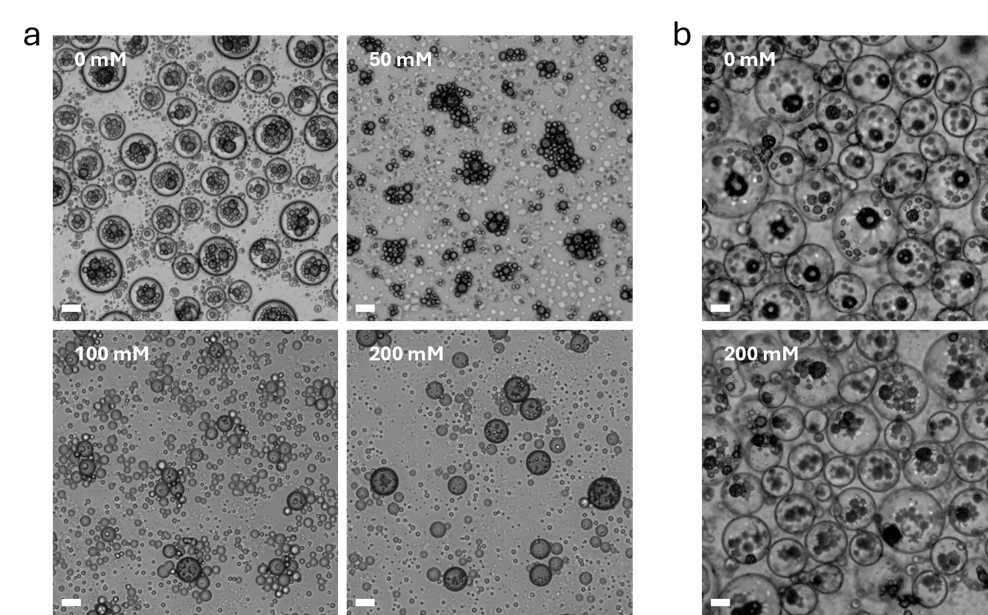


**Supplementary Figure 7.** Dispersions of **MCD** (**a**) and **NCV** (**b**) containing NaCl at different concentrations (0-200 mM), analyzed by bright field microscopy. Composition: PDDA/PAH/ATP, 20/10/12.5 mM; PTA, 0 or 3 mM. Scale bars, 20 μm.


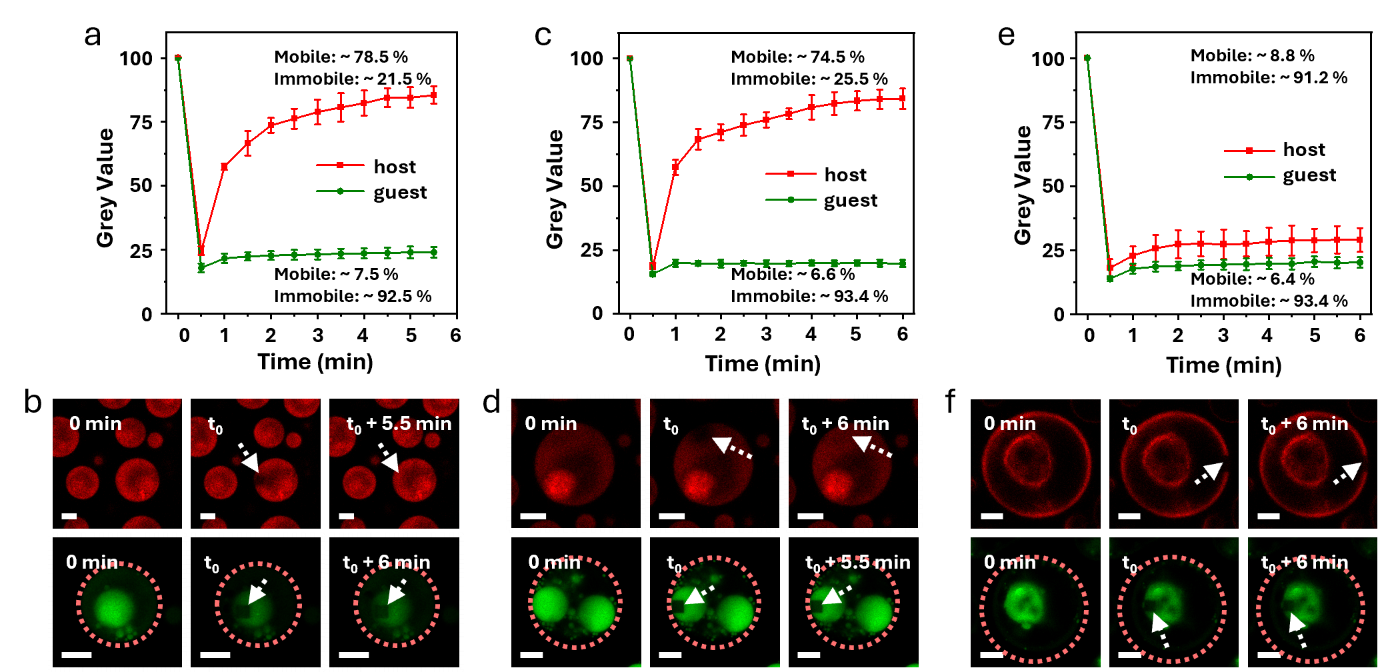


**Supplementary Figure 8.** (**a**), (**c**) and (**e**) are FRAP profiles of MCDs prepared at pH 4 and 6.5 and NCVs prepared at pH 6.5, respectively. (**b**), (**d**) and (**f**) are the corresponding time-dependent CLSM images showing the bleaching and recovery of fluorescence in the host (upper) and guest domains (bottom) of MCD and NCV. Mobile and immobile fractions are indicated in the recovery profiles to quantitatively describe the viscoelastic differences between the domains. The guest domain of the MCDs exhibits higher viscoelasticity than the host domain. After PTA-induced reconfiguration, the PDDA-rich host domain in the NCVs displays increased viscoelasticity. Composition: RITC-PDDA/FITC-PAH/ATP, 20/10/12.5 mM; PTA, 0 or 3 mM. Scale bars, 20 μm.


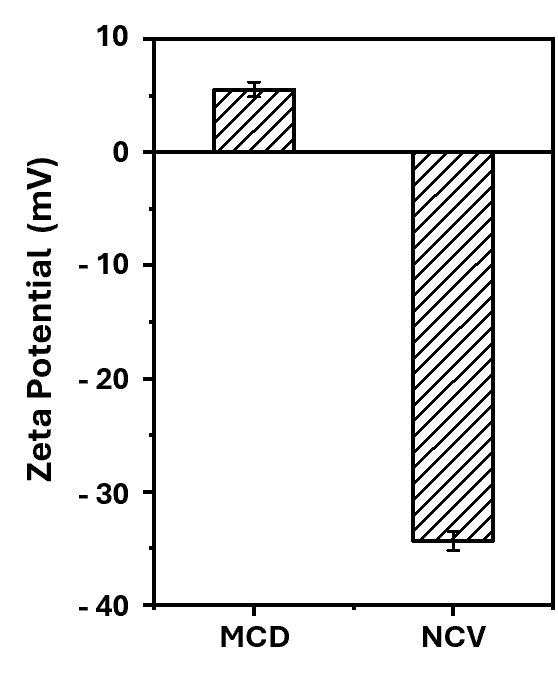


**Supplementary Figure 9.** Bar charts showing zeta potential of MCDs (+ 5.5 mV) and NCVs (-34.3 mV) at pH ca. 6.5. Composition: PDDA/PAH/ATP, 20/10/12.5 mM; PTA, 0 (MCD) or 3 mM (NCV). Error bars: n = 3, mean value ± SD.


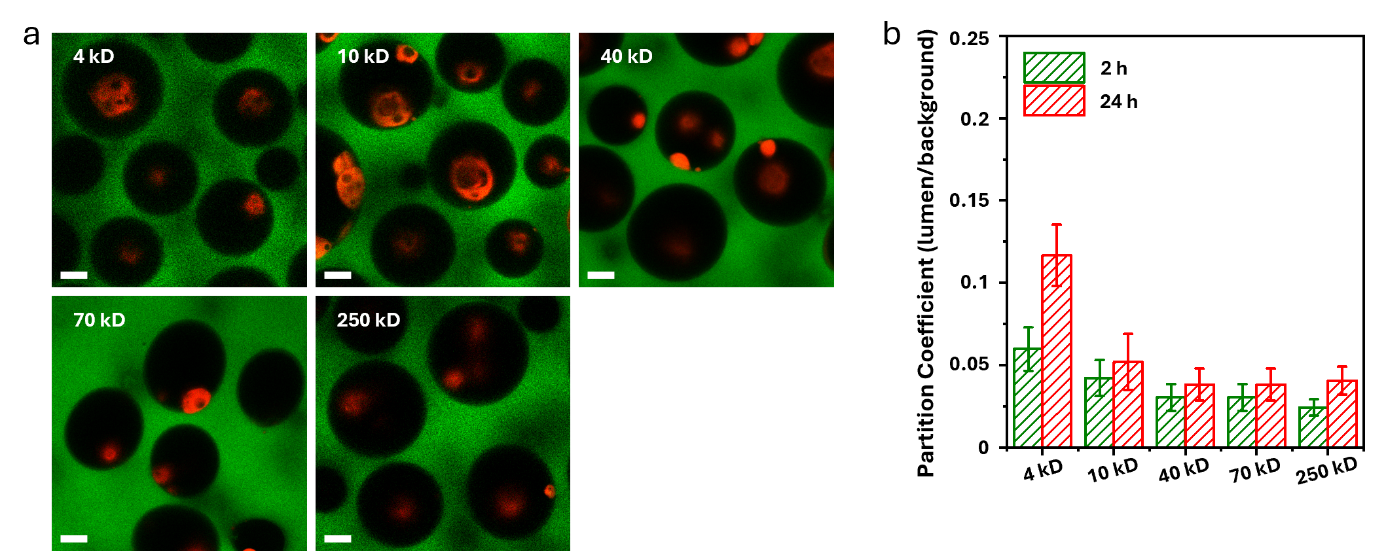


**Supplementary Figure 10.** Permeability of NCV constructs. (**a**) CLSM images of NCVs incubated with FITC-dextran of different molecular weights (4, 10, 40, 70, 250 kDa) for 24 h. Composition: PDDA/RITC-PAH/ATP, 20/10/12.5 mM; PTA, 3 mM; FITC-dextran, 0.001 mg/ml. (**b**) Partition coefficients of FITC-dextran, calculated as fluorescence intensity ratios (grey values) inside versus outside NCVs. Error bars: n ≥ 20 from over 3 CLSM images. Scale bars, 20 μm.


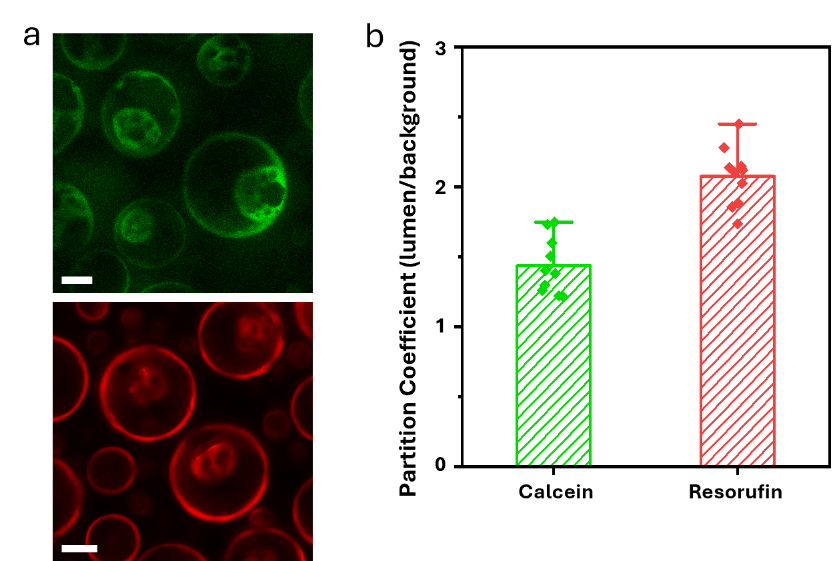


**Supplementary Figure 11.** Permeability of NCV constructs. (**a**) CLSM images of NCVs incubated with calcein (upper) and resorufin (bottom). Composition: PDDA/ PAH/ATP, 20/10/12.5 mM; PTA, 3 mM; dyes, 0.005 mg/ml. (**b**) Partition coefficients of calcein and resorufin dyes, calculated as fluorescence intensity ratios (grey values) inside versus outside NCVs. Error bars: n ≥ 20 from over 3 CLSM images. Scale bars, 20 μm.


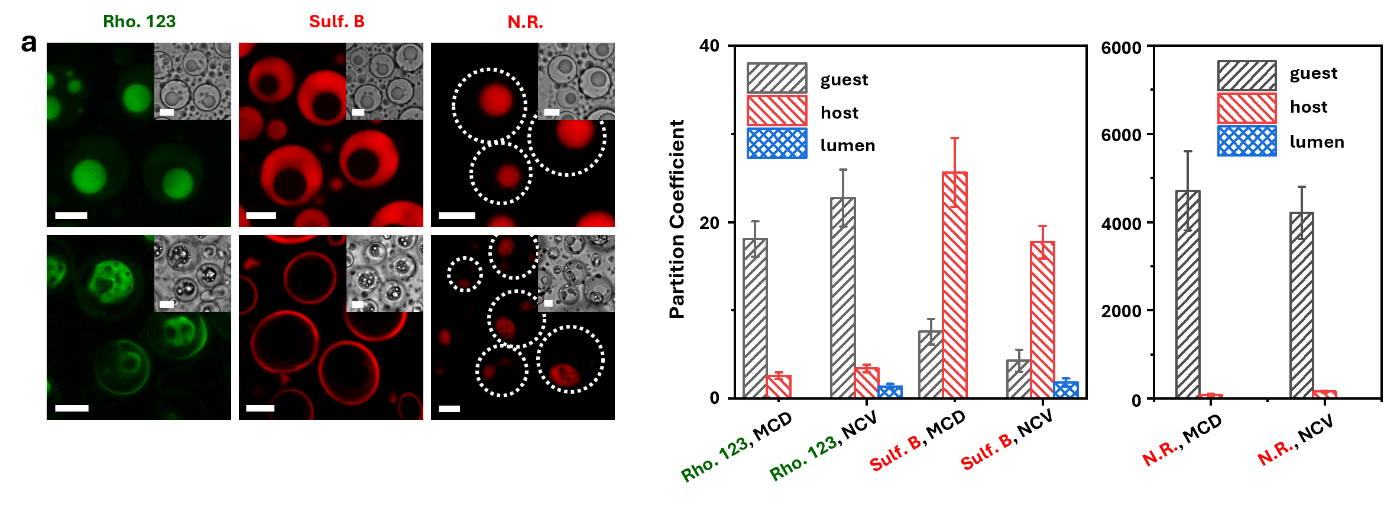


***Supplementary Figure 12.*** *(****a****)* *CLSM images of MCDs (top) and NCVs (bottom) loaded with positively charged rhodamine 123 (Rho. 123, left), negatively charged sulforhodamine B (Sulf. B, middle), and neutral Nile red (N.R., right). White dashed circles delineate the boundaries of the host domains of MCDs and NCVs. Insets are the corresponding bright-field images. (****b****) Bar charts showing partition coefficients of dyes in MCDs and NCVs, calculated as fluorescence intensity ratios (grey values) of guest (inner phase), host domains (outer phase) or lumen of NCV versus the continuous dilute phase using Image J software. Composition: PDDA/PAH/ATP, 20/10/12.5 mM; PTA, 0 or 3 mM. Fluorescent dyes, 0.005 mg/ml. pH value, ca. 6.5. Error bars: n ≥ 20 from over 3 CLSM images. Scale bars, 20 μm.*


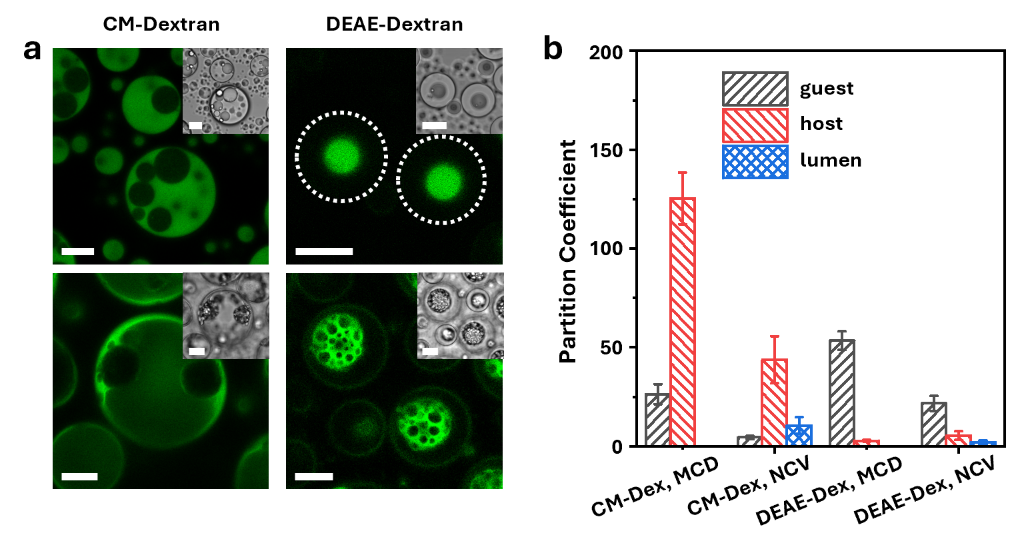


***Supplementary Figure 13.*** *Distribution of polysaccharides within MCD and NCV constructs. (****a****) CLSM images of MCD (upper) and NCV (bottom) loaded with FITC-labelled CM-dextran (left) and DEAE-dextran (right). White dashed circles delineate the boundaries of the host domain of MCDs. Insets are the corresponding bright-field images. (****b****) Bar charts showing partition coefficients of FITC-polysaccharides in MCDs and NCVs, calculated as fluorescence intensity ratios (grey values) of guest (inner phase), host domains (outer phase) or lumen of NCV versus the continuous dilute phase using Image J software. Upon reconfiguration of MCDs into NCVs, significant accumulation of FITC–CM-dextran was observed within the inner space of the NCV host domain. Composition: PDDA/PAH/ATP, 20/10/12.5 mM; PTA, 0 or 3 mM. FITC-labelled polysaccharides, 0.005 mg/ml. pH value, ca. 6.5. Error bars: n ≥ 20 from over 3 CLSM images. Scale bars, 20 μm.*


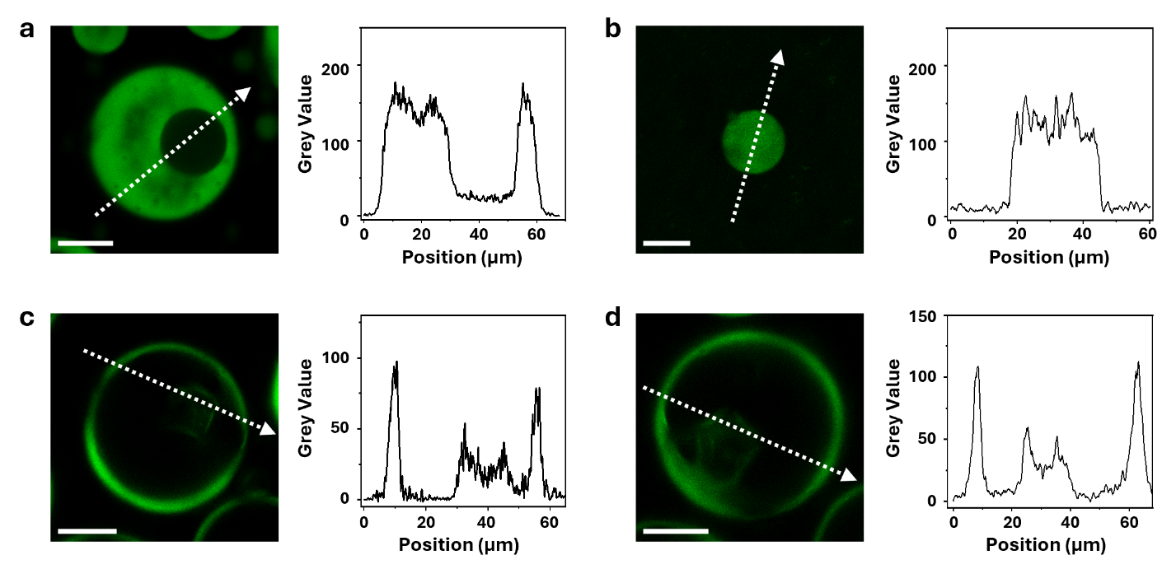


**Supplementary Figure 14.** (**a**) and (**b**) are CLSM images (left) of FITC-GOx-loaded MCD constructs dispersed in 0 (**a**) and 100 mM (**b**) NaCl solution. (**c**) and (**d**) CLSM images (left) of FITC-GOx-loaded NCV constructs dispersed in 0 (**c**) and 100 mM (**d**) NaCl solution. From (**a**) to (**d**), right is the corresponding line profile indicating the distribution of the FITC-labelled GOx. The host domain of the MCD construct is dissociated in 100 mM NaCl solution leading to translocation of the FITC-labelled GOx into the guest domain. In contrast, the spatial organization of FITC-labelled GOx in the NCV construct stays consistent in different concentrations of NaCl solutions. Composition: PDDA/PAH/ATP, 20/10/12.5 mM; PTA, 0 or 3 mM. FITC-labelled GOx, 0.01 mg/ml. pH value are all set to ca. 6.5. Error bars: n ≥ 20 from over 3 CLSM images. Scale bars, 20 μm.


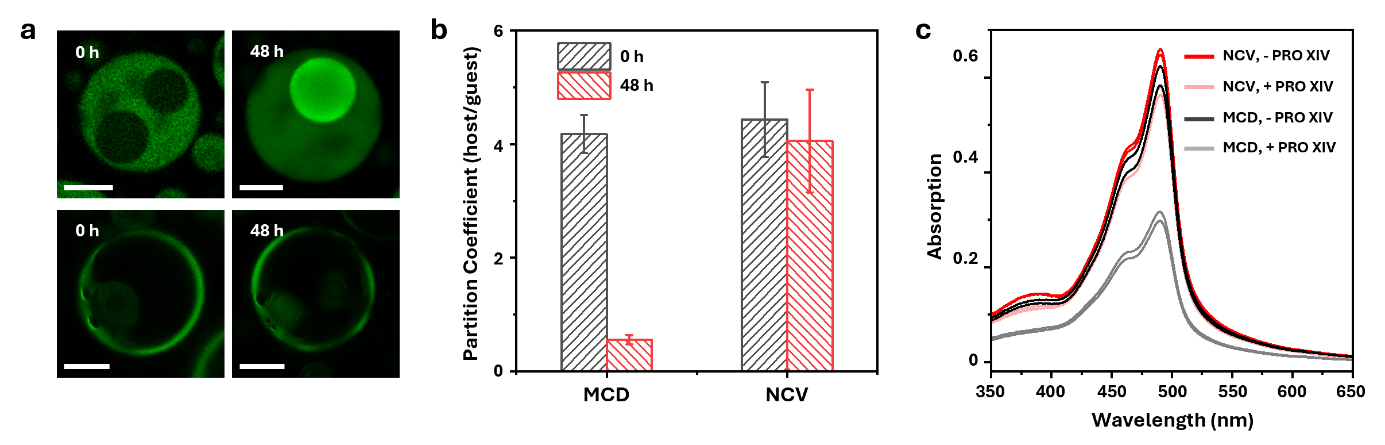


**Supplementary Figure 15.** (**a**) Time-dependent CLSM images of MCD (upper) and NCV (bottom) constructs loaded with FITC-labelled GOx and incubated in protease XIV (**PRO XIV**) solution. Composition: PDDA/PAH/ATP, 20/10/12.5 mM; PTA, 0 or 3 mM. Protease XIV, 3.5 IU/ml. FITC-labelled GOx, 0.01 mg/ml. pH value, *ca.* 6.5. (**b**) Bar charts showing the partition coefficients of FITC-labelled GOx within the MCDs and NCVs, calculated as the fluorescence intensity (grey value) ratios of the host versus guest domains. In contrast to membranous and reinforced NCVs, more significant spatial translocation of fluorescence was observed within the MCDs due to proteolytic degradation of FITC-GOx. (**c**) UV/Vis profiles showing *o*-PD oxidation induced by GOx/HRP-loaded NCV and MCD constructs in the presence and absence of protease XIV. Protease XIV, 0 or 3.5 IU/ml. Substrate: glucose 10 mM; *o*-PD 20 mM. GOx/HRP-loaded NCVs retained higher catalytic activity than MCD constructs after protease XIV treatment. Two repeated samples in each group. Error bars: n ≥ 20 from over 3 CLSM images. Scale bars, 20 μm.


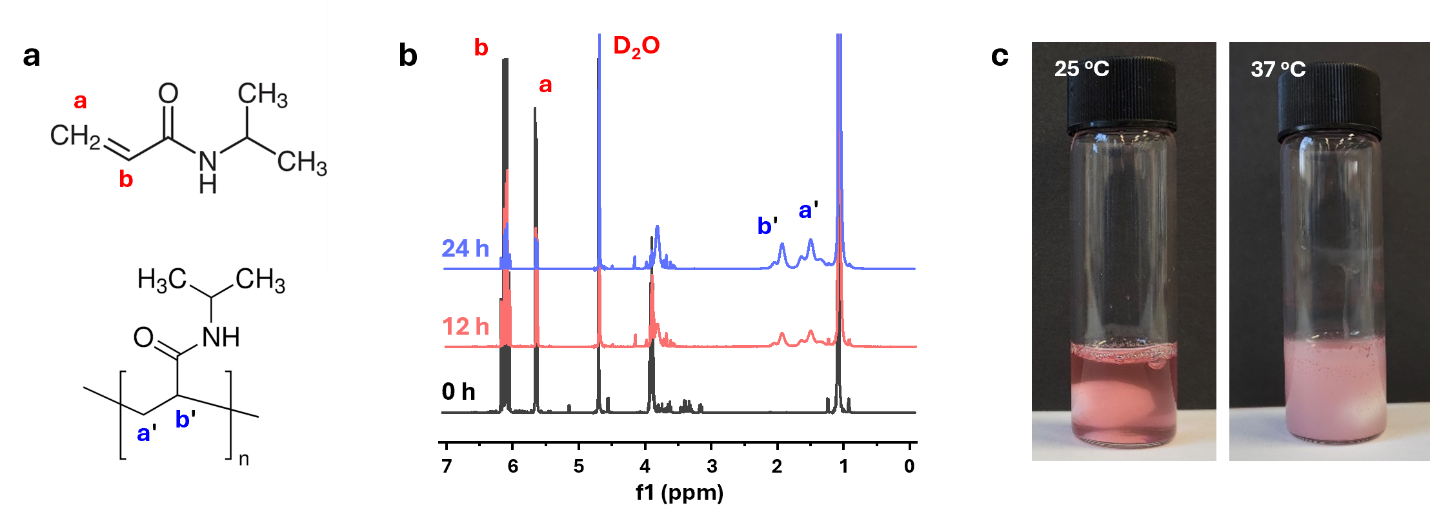


**Supplementary Figure 16.** (**a**) Structures of NIPAAm (top) and PNIPAAm (bottom). (**b**) Time-dependent ¹H NMR spectra of a reaction mixture containing GOx (*ca.* 20 unit/ml), GNPs (20 nm, OD_520_ = 1), NIPAAm (20 mg/ml), and glucose (10 mM) incubated under N_2_ for 0-24 h (pH 7.0). Characteristic ^1^H NMR signals at 1.94 and 1.50 ppm increased during polymerization. (**c**) Photographs of the reaction mixture at 25 and 37 ^o^C after ca. 24 h, showing PNIPAAm dissolution at 25 ^o^C and phase transition to aggregates at 37 ^o^C.


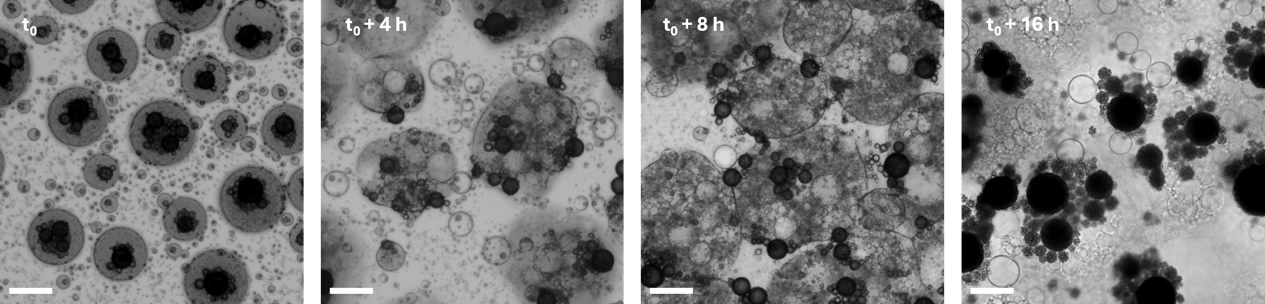


**Supplementary Figure 17**. Time-dependent bright field images of MCD constructs undergoing *in situ* polymerization of NIPAAm upon glucose addition. PDDA/PAH/ATP, 10/10/12.5 mM; NIPAAm, *ca.* 25 mg/ml. Scale bars, 20 µm.


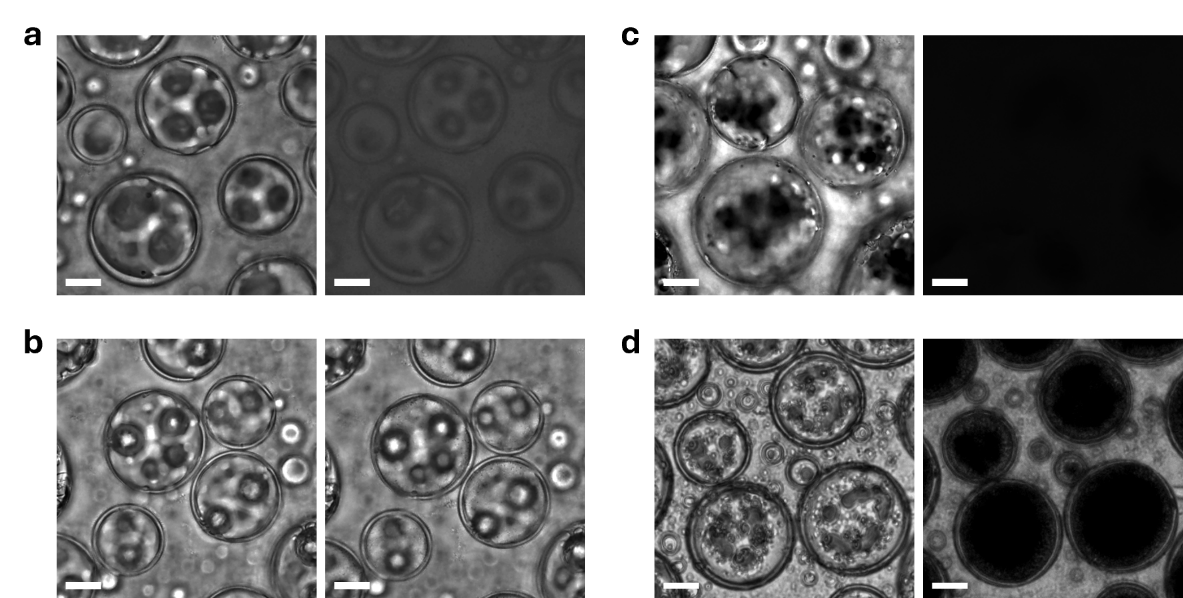


**Supplementary Figure 18** Evaluation of PNIPAAm synthesis within GOx-loaded (**a**,**b**) and GOx/GNPs-loaded (**c**,**d**) NCV constructs by incubation at 25 (left) and 37 ^o^C (right) and imaging the constructs using bright field CLSM. Images were recorded before (**a**,**c**) and after (**b**,**d**) a rinsing process to remove PNIPAAm in the external environment. Composition: PDDA/PAH/ATP, 20/10/12.5 mM; PTA, 3 mM; GOx, 0.05 mg/ml; GNPs (20 nm), OD₅₂₀ = 0 or 1. Substrate: NIPAAm, 20 mg/ml; glucose, 10 mM. Scale bars, 20 µm.


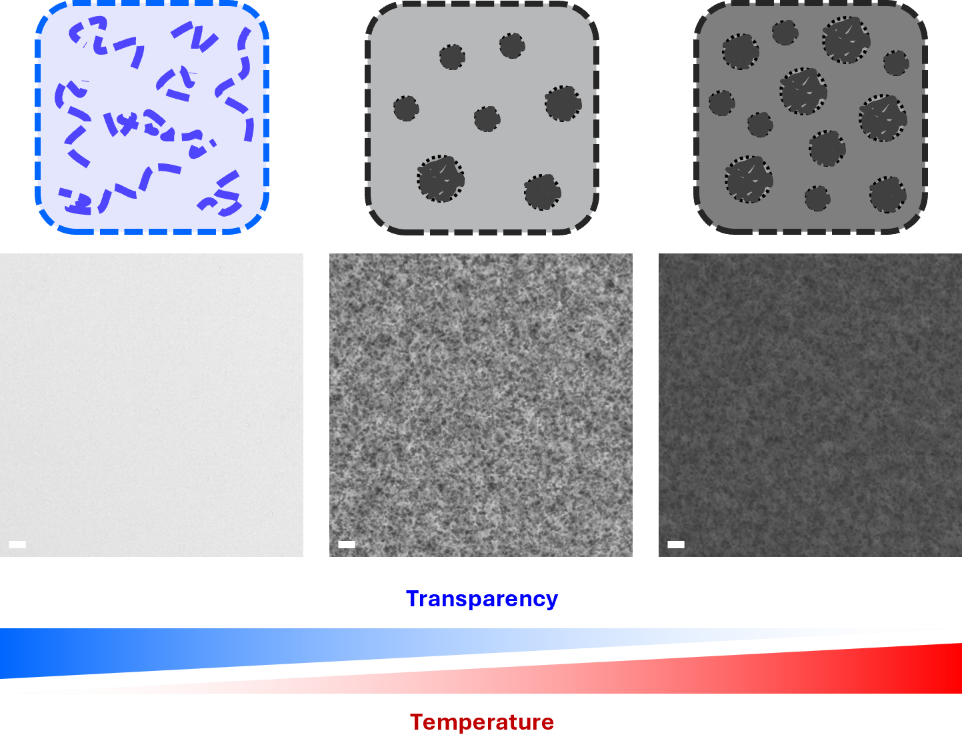


**Supplementary Figure 19.** Temperature-dependent transparency change of PNIPAAm solutions. A transparent PNIPAAm solution (Mw = 10 kDa, ca. 10 mg/ml, R.T.) sealed in a glass channel (thickness 0.1 mm) becomes dark upon heating to 37 °C due to the formation of aggregates, which reduce light transmission by reflection and scattering. Images were recorded by inverted bright-field microscopy. Scale bars, 20 µm.


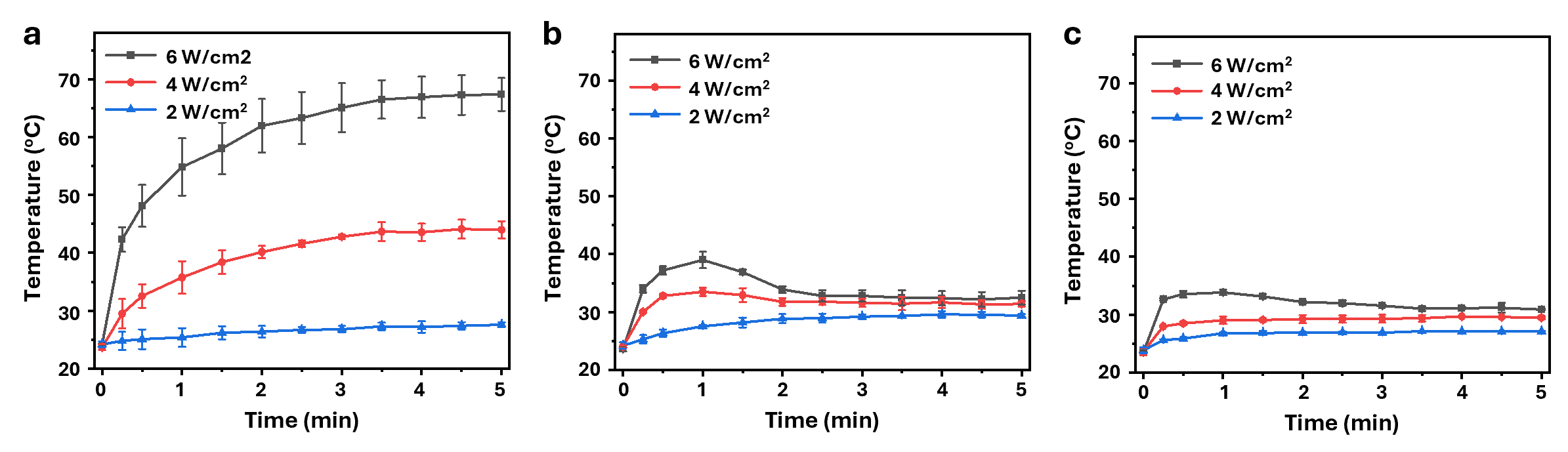


**Supplementary Figure 20.** Time-dependent photothermal transitions of NCVs containing different levels of synthesized PNIPAAm. PNIPAAm content is regulated by initiating NIPAAm polymerization at 0 (**a**), 25 (**b**), or 50 (**c**) mg/ml. Photothermal transitions are induced by NIR irradiation (808 nm, 2-6 W/cm^2^) and monitored with a thermal camera. Error bars: n = 3 (repeated groups of sample), mean value ± SD.


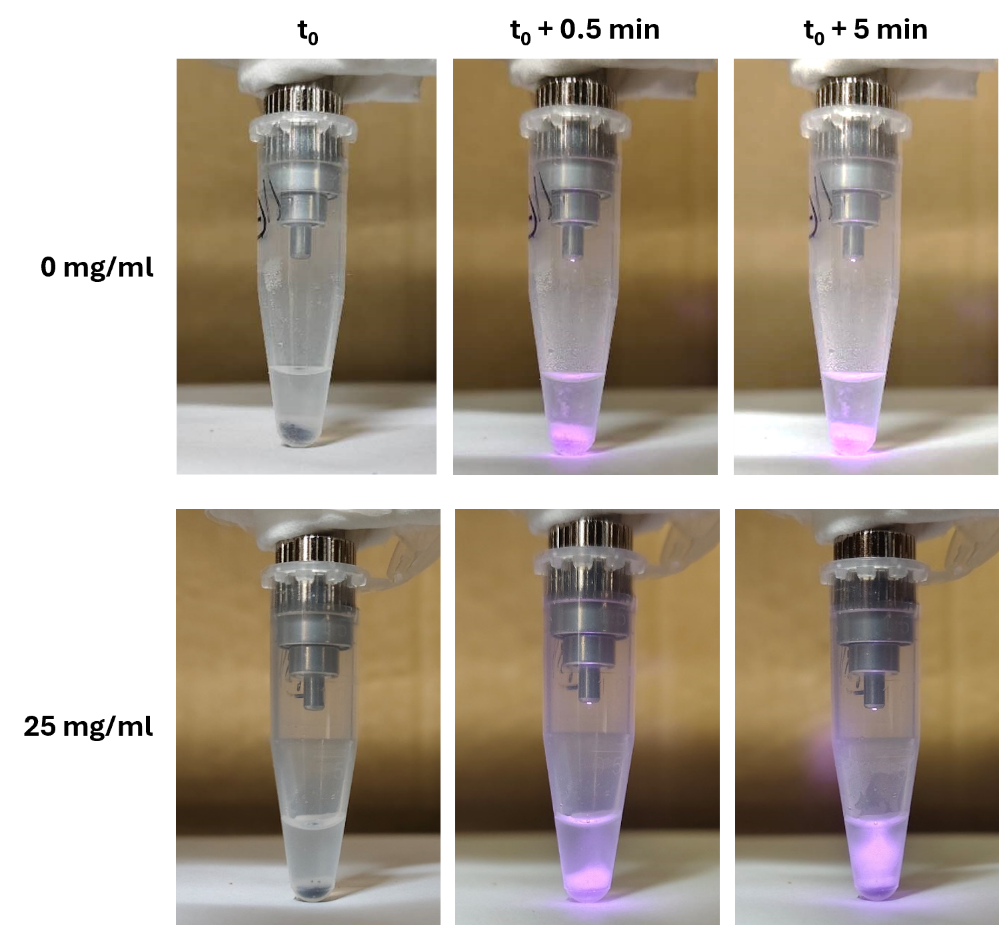


**Supplementary Figure 21.** Time-dependent photographs of NCVs with or without synthesized PNIPAAm under NIR irradiation. NCV dispersions (PDDA/PAH/ATP, 20/10/12.5 mM; PTA, 3 mM; GOx, 0.05 mg mL⁻¹; GNPs, OD_520_ = 1) are prepared with NIPAAm (0 or 25 mg/ml) and glucose (10 mM). Upon irradiation (808 nm, 6 W/cm^2^), light transmission through GNP-loaded NCVs lacking PNIPAAm remains constant, whereas PNIPAAm-containing NCVs gradually scatter light due to formation of PNIPAAm aggregates (bottom).


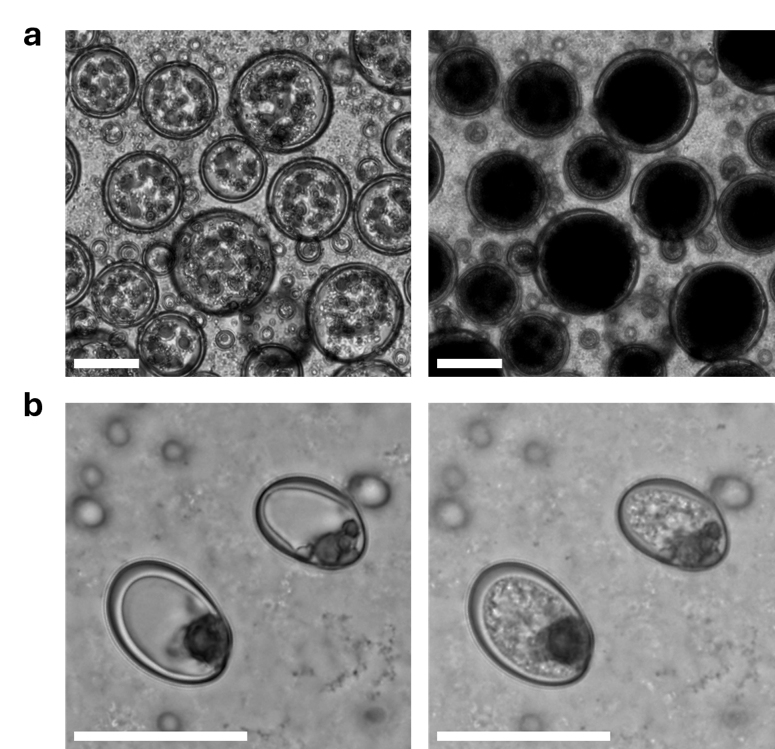


**Supplementary Figure 22.** NCV constructs containing spatially confined PNIPAAm. (**a**) Inverted optical microscopy and (**b**) bright-field CLSM images of NCVs incubated at 25 °C (left) or 37 °C (right). Background components are removed by rinsing NCVs with DI water after PNIPAAm synthesis. Composition: PDDA/PAH/ATP, 20/10/12.5 mM; PTA, 3 mM; GOx, 0.05 mg/ml; GNPs (20 nm), OD₅₂₀ = 1. Substrate: NIPAAm, 20 mg/ml; glucose, 10 mM. Scale bars, 20 µm.


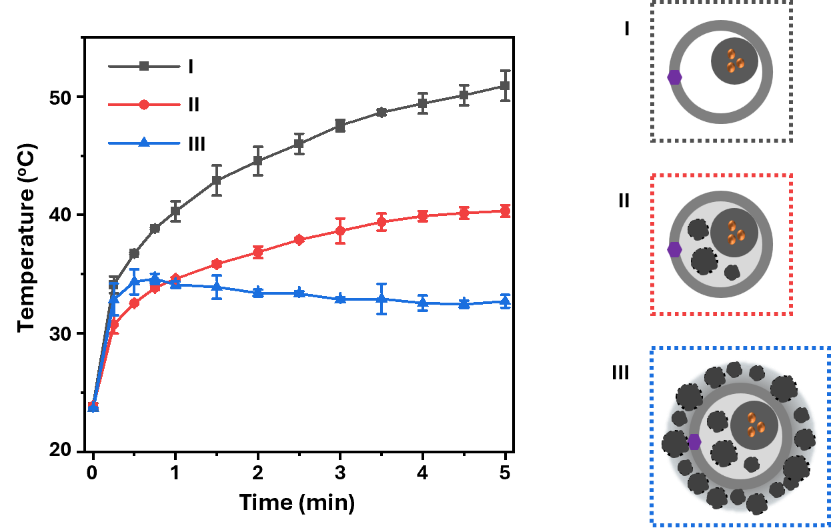


**Supplementary Figure 23.** Photothermal transition of NCVs with differently spatial confined PNIPAAm. NCVs are prepared under three conditions: Group I, NIPAAm 0 mg/ml, glucose 10 mM; Groups II and III, NIPAAm 25 mg/ml, glucose 10 mM. Reaction mixtures were incubated at R.T. for *ca.* 24 h for polymerization. In Group II, background PNIPAAm was removed by rinsing with DI water. Photothermal transitions were induced by NIR irradiation (808 nm, 6 W/cm^2^) and monitored using a thermal camera. Composition: PDDA/PAH/ATP, 20/10/12.5 mM; PTA, 3 mM; GOx, 0.05 mg/ml; GNPs (20 nm), OD₅₂₀ = 1. Substrate: NIPAAm, 20 mg/ml; glucose, 10 mM. Error bars: n = 3 (repeated groups of sample), mean value ± SD.


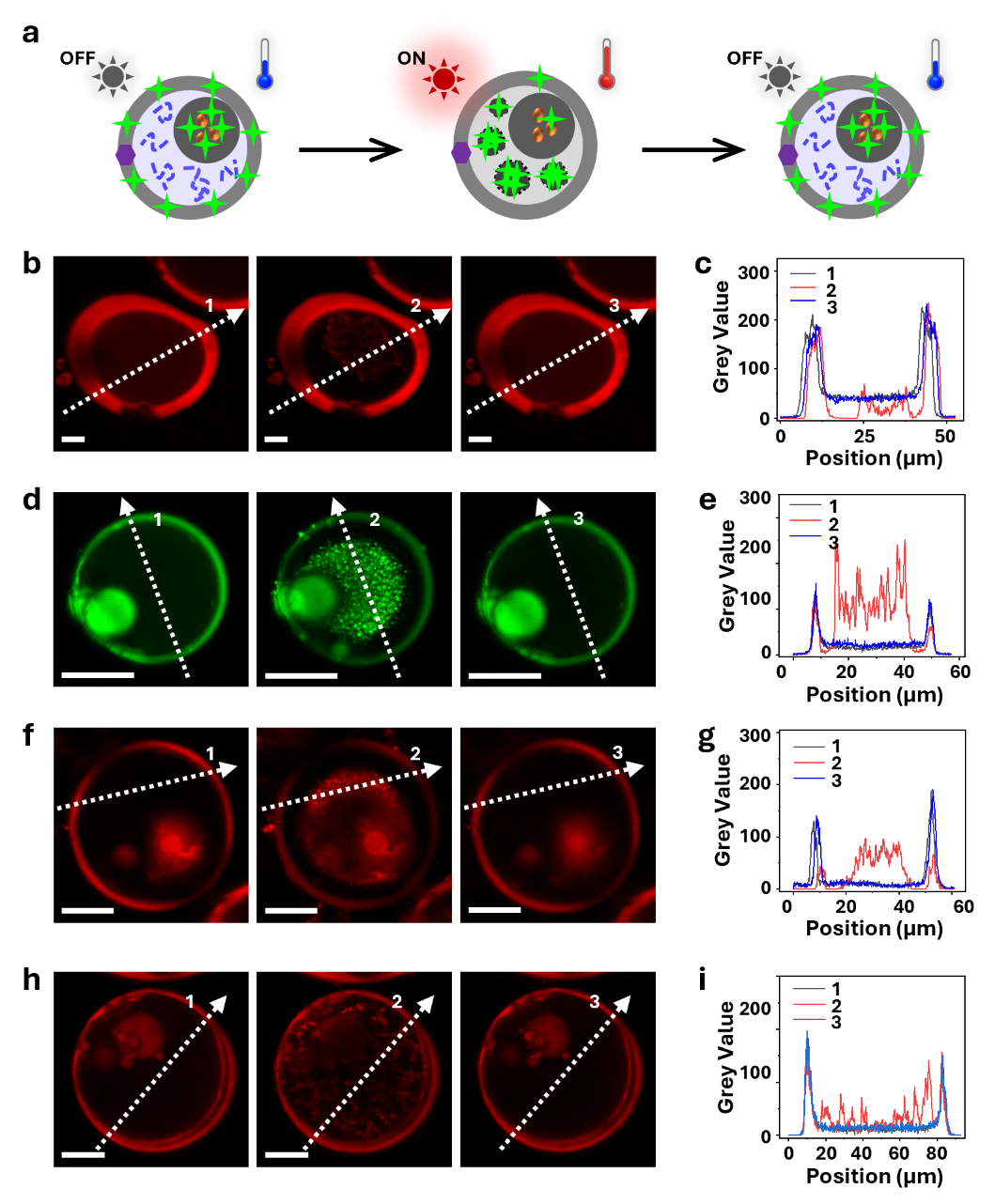


**Supplementary Figure 24.** Spatiotemporally regulated organization of cargoes within NCV constructs. (a) Scheme illustrating fluorescent dyes (green stars) initially confined within the NCV membrane translocate into PNIPAAm aggregates formed upon photothermal heating under NIR irradiation (808 nm). When the light is switched off, the aggregates dissociate and the dyes diffuse back to their original sites. (b), (d), (f) and (h) are CLSM images of PNIPAAm-containing NCV constructs stained by resorufin, rhodamine 123, rhodamine 6g, and Nile red, respectively, irradiated by NIR light (808 nm) at 0 (left), 6 (middle) and 0 (right) W/cm^2^ for 2 min. (c), (e), (g) and (i) are line profiles indicating the distribution of fluorescent dyes within the NCV constructs. Composition: PDDA/PAH/ATP, 20/10/12.5 mM; PTA, 3 mM; NIPAAm, 25 mg/ml. pH ca. 6.5. Scale bars, 10 μm.


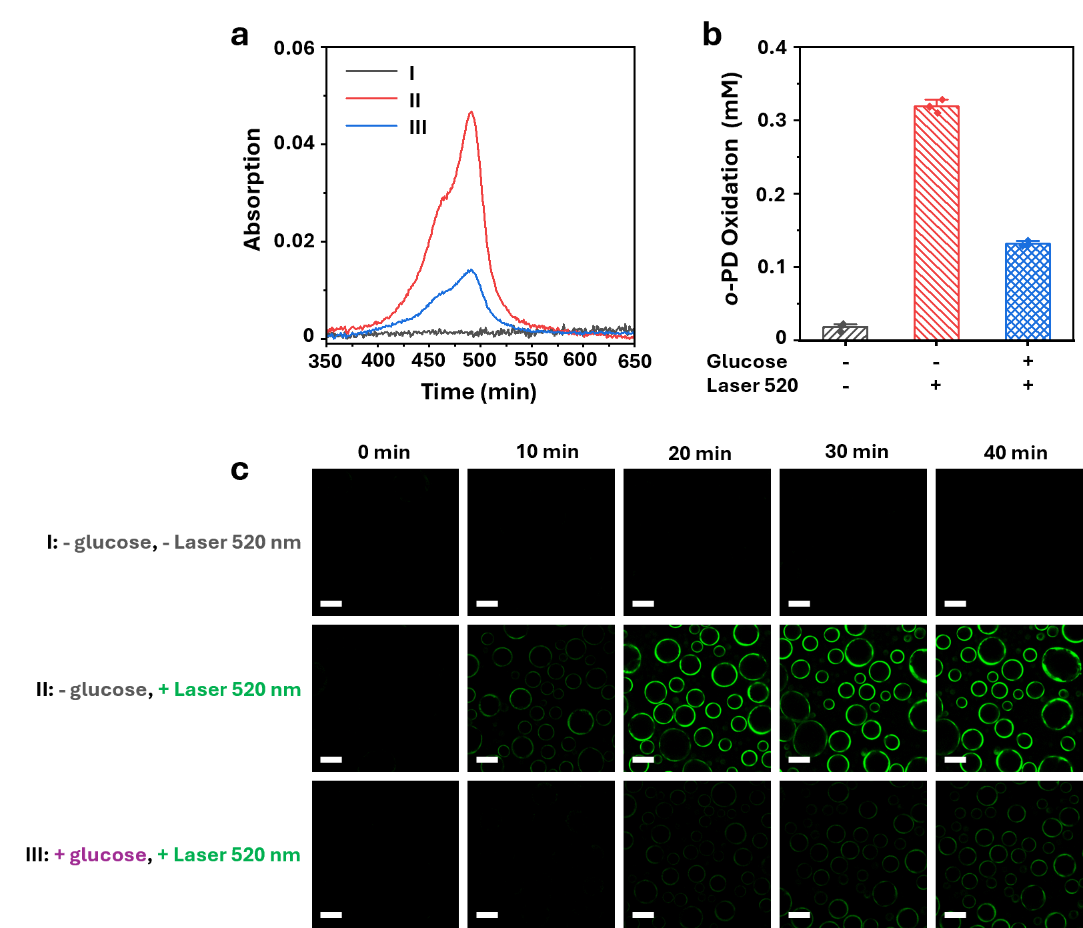


**Supplementary Figure 25.** GNP-mediated photocatalytic *o*-PD oxidation within NCVs. (**a**) Absorption spectra and (**b**) bar charts showing o-PD oxidation under different conditions: Group I, glucose 0 mM, laser 520 nm, 0 W/cm^2^; Group II, glucose 0 mM, laser 520 nm, 0.1 W/cm^2^; Group III, glucose 10 mM, laser 520 nm, 0.1 W/cm^2^. (c) Time-dependent CLSM images of NCVs showing green fluorescence from DAP formed during photocatalytic o-PD oxidation. NCV composition: PDDA/PAH/ATP, 20/10/12.5 mM; PTA, 3 mM; GOx, 0.05 mg/ml; GNPs (20 nm), OD₅₂₀ = 1; *o*-PD, 10 mM. Error bars: n = 3 (repeated groups of sample), mean value ± SD in (b). Scale bars, 20 µm.


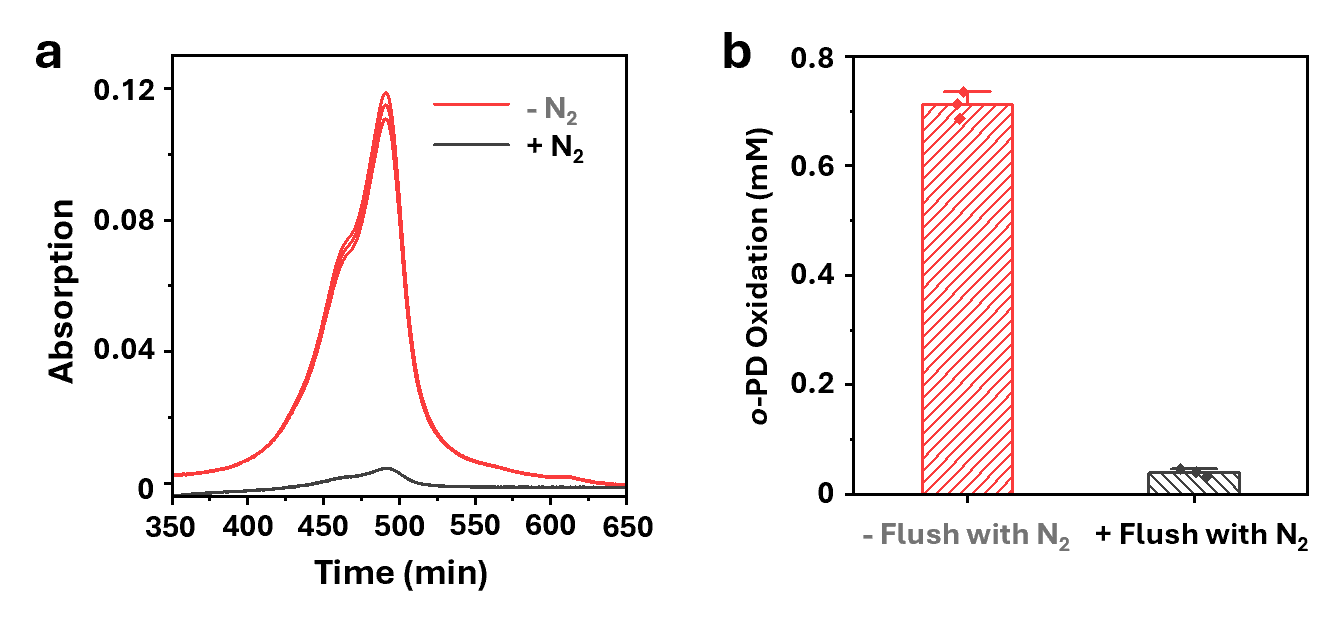


**Supplementary Figure 26.** (**a**) UV/Vis profiles and (**b**) bar charts showing O_2_-dependent o-PD oxidation within GOx/GNPs-loaded NCVs. The O_2_ level is regulated by flushing the reaction mixtures with N_2_ for 0 and 30 min. *o*-PD oxidation is induced by irradiation with NIR light (808 nm, *ca.* 6 W/cm^2^). Composition: PDDA/PAH/ATP, 20/10/12.5 mM; PTA, 3 mM; GOx, 0.05 mg/ml; GNPs (20 nm), OD_520_ = 1. Substrate concentration: o-PD 10 mM; glucose, 0 mM. Error bars: n = 3 (repeated group of sample), mean value ± SD.


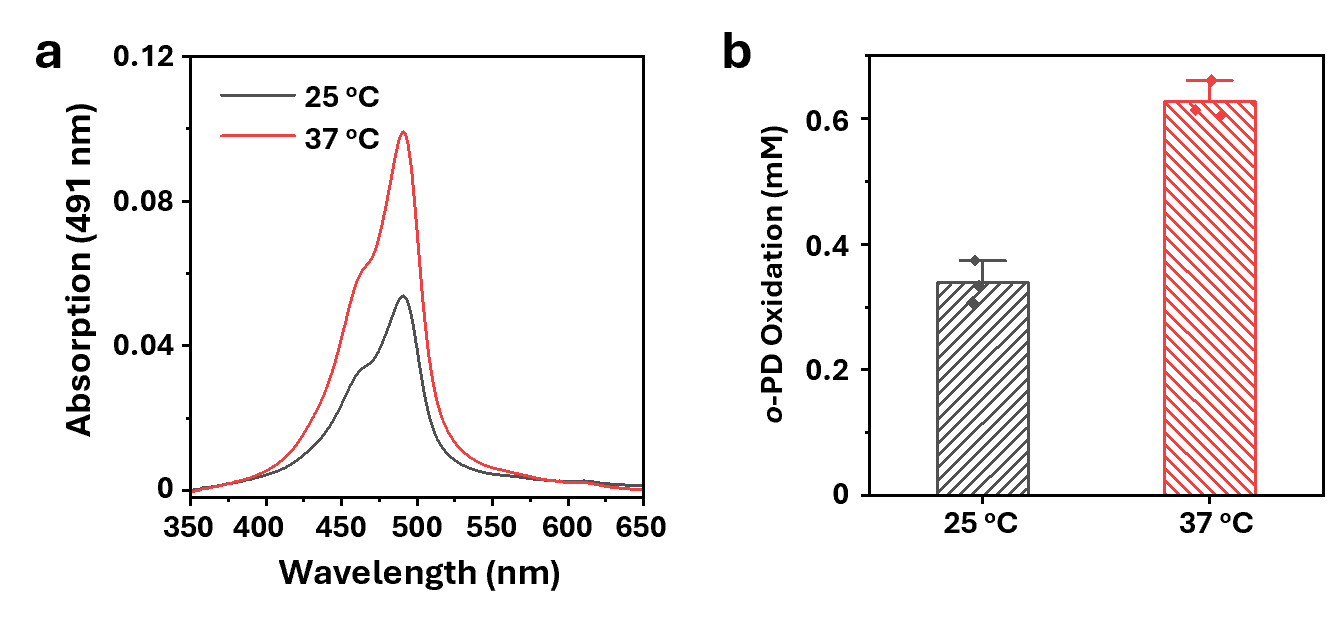


**Supplementary Figure 27.** (**a**) UV/Vis profiles and (**b**) bar charts showing temperature-dependent o-PD oxidation in GOx/GNPs-loaded NCVs. *o*-PD oxidation is induced by irradiation with NIR light (808 nm, *ca.* 6 W/cm^2^). Reaction time: *ca.* 0.5 h. Composition: PDDA/PAH/ATP, 20/10/12.5 mM; PTA, 3 mM; GOx, 0.05 mg/ml; GNPs (20 nm), OD₅₂₀ = 1. Substrate concentration: *o*-PD, 10 mM; glucose, 0 mM. Error bars: n = 3 (repeated group of sample), mean value ± SD.
